# Gadolinium-Based NMR Spin Relaxation Measurements of Near-Surface Electrostatic Potentials of Biomolecules

**DOI:** 10.1021/jacs.4c04433

**Published:** 2024-07-19

**Authors:** Binhan Yu, Nicolas Bolik-Coulon, Atul K. Rangadurai, Lewis E. Kay, Junji Iwahara

**Affiliations:** † Department of Biochemistry & Molecular Biology, Sealy Center for Structural Biology & Molecular Biophysics, University of Texas Medical Branch, Galveston, Texas 77555-1068, United States; ‡ Department of Molecular Genetics, University of Toronto, Toronto, Ontario M5S 1A8, Canada; § Department of Chemistry, University of Toronto, Toronto, Ontario M5S 3H6, Canada; ∥ Department of Biochemistry, University of Toronto, Toronto, Ontario M5S 3H6, Canada; ⊥ Program in Molecular Medicine, Hospital for Sick Children Research Institute, Toronto, Ontario M5G 0A4, Canada

## Abstract

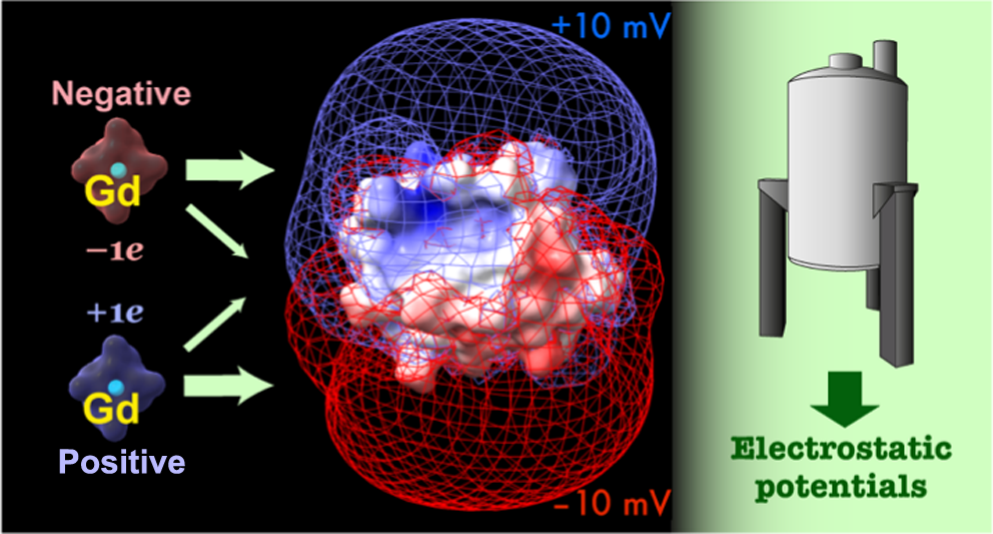

NMR spectroscopy is an important tool for the measurement of the electrostatic properties of biomolecules. To this point, paramagnetic relaxation enhancements (PREs) of ^1^H nuclei arising from nitroxide cosolutes in biomolecular solutions have been used to measure effective near-surface electrostatic potentials (ϕ_ENS_) of proteins and nucleic acids. Here, we present a gadolinium (Gd)-based NMR method, exploiting Gd chelates with different net charges, for measuring ϕ_ENS_ values and demonstrate its utility through applications to a number of biomolecular systems. The use of Gd-based cosolutes offers several advantages over nitroxides for ϕ_ENS_ measurements. First, unlike nitroxide compounds, Gd chelates enable electrostatic potential measurements on oxidation-sensitive proteins that require reducing agents. Second, the large electron spin quantum number of Gd (7/2) results in notably larger PREs for Gd chelates when used at the same concentrations as nitroxide radicals. Thus, it is possible to measure ϕ_ENS_ values exclusively from + and – charged compounds even for highly charged biomolecules, avoiding the use of neutral cosolutes that, as we further establish here, limits the accuracy of the measured electrostatic potentials. In addition, the smaller concentrations of cosolutes required minimize potential binding to sites on macromolecules. Fourth, the closer proximity of the paramagnetic center and charged groups within Gd chelates, in comparison to the corresponding nitroxide compounds, enables more accurate predictions of ϕ_ENS_ potentials for cross-validation of the experimental results. The Gd-based method described here, thus, broadens the applicability of studies of biomolecular electrostatics using solution NMR spectroscopy.

## Introduction

Electrostatics often play an important role in molecular recognition, enzyme catalysis, and phase separation and, thus, in controlling biomolecular function.^1−5^ Additionally, charge effects must be understood and properly taken into account for the successful design of proteins and in the development of potent biopharmaceuticals.^6−9^ For a macromolecule in solution, the electrostatic potential depends not only on ionizable functional groups within the molecule but also on the concentrations and spatial distributions of mobile ions (e.g., K^+^, Na^+^, Cl^–^) surrounding the molecule.^10^ These ions dampen electric fields, influencing the stabilities of the dissolved biomolecules as well as the kinetics and thermodynamics of their binding to partners.^1,5,11,12^

Insights into biomolecular electrostatics have been primarily obtained through computation based on static three-dimensional (3D) structures. Computer programs such as APBS^13,14^ and DelPhi^15,16^ are used to numerically solve the Poisson–Boltzmann equation,^17^ taking mobile ions into consideration and outputting calculated electrostatic potentials on grid points in a 3D space encompassing the biomolecule of interest. Such electrostatic potentials, as computed from structures, are useful for rigid molecules. However, many proteins and nucleic acids contain conformationally flexible segments, for which accurate structural ensembles are usually unavailable. For example, approximately 70% of human proteins have intrinsically disordered regions (IDRs) comprised of 30 residues or longer.^18^ The charge features of IDRs can be critically important for controlling intra- or intermolecular contacts with targets, with these interactions modulated by post-translational modifications that alter charge.^5,18,19^ To date, an accurate quantification of the electrostatic properties of flexible regions of biomolecules has been largely elusive.

**Figure 1:**
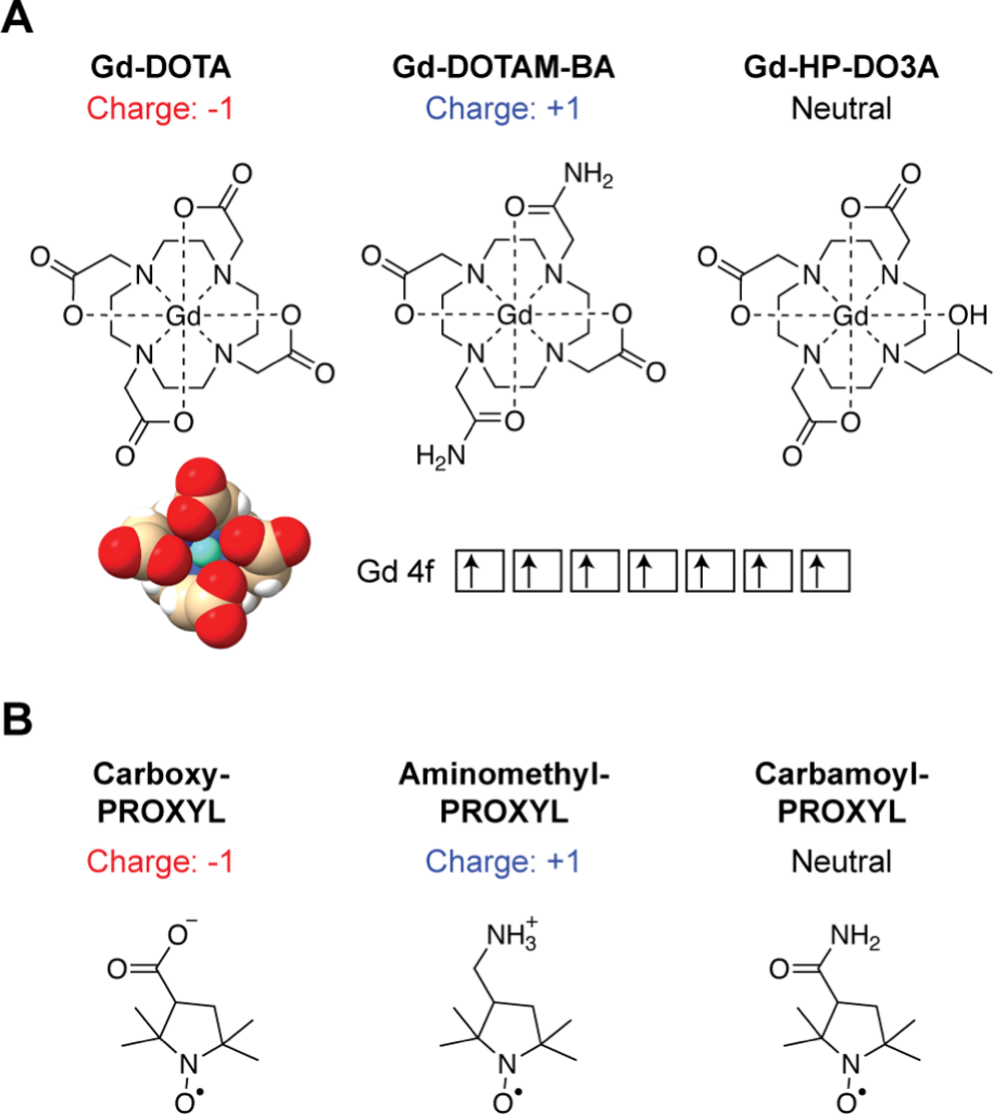
Paramagnetic cosolutes used to measure electrostatic potentials of biomolecules in the current study. (A) Gadolinium (Gd) chelates. Each chelate has 7 unpaired electrons in 4f orbitals of the Gd atom (the electron spin quantum number *S* = 7/2). A crystal structure of Gd-DOTA (Cambridge Structural Database JOPJIH01)^29^ is also shown. (B) Nitroxide compounds. Each PROXYL derivative has 1 unpaired electron (*S* = 1/2) as a stable radical. Although both charged and neutral paramagnetic compounds are shown here, the use of neutral compounds is not recommended because it reduces the accuracy of measured electrostatic potentials (see text).

An important advance in the measurement of electrostatic potentials of biomolecules in solution is the recent emergence of an NMR method that allows access to such information without the requirement of any structural data.^20^ This method utilizes paramagnetic relaxation enhancements (PREs) of protons within the molecule of interest that arise from added nitroxide cosolutes of different charges (Figure 1) whose spatial positions are differentially biased by the charge distribution of the molecule. Using this NMR method, an effective near-surface electrostatic potential (ϕ_ENS_) representing a local average of the electrostatic potentials in a region near the observed ^1^H nucleus, can, in principle, be measured simultaneously for all of the biomolecular protons that are sufficiently close (<∼15 Å) to the solvent.^20^ Because the ϕ_ENS_ method does not require any structural information, its applicability extends to conformationally flexible systems, including IDRs of proteins.^21^ For example, the ϕ_ENS_ method has been applied to map per-residue surface electrostatic potentials of the positively charged 103 residue carboxyl-terminus of the 702 residue RNA-binding protein CAPRIN1 along its phase-separation trajectory.^22,23^ This NMR method has also been used to investigate the electrostatic properties of the unfolded drk SH3 domain^24^ and the disordered Pmel17 repeat domain,^25^ as well as electrostatic interactions between a number of different proteins and their conformationally flexible ligands.^26^

Although the nitroxide cosolute-based NMR method for the direct measurement of electrostatic potentials is powerful, there are a number of practical issues that hinder its broad applicability. For example, proteins containing cysteine thiols typically require a reducing agent (e.g., dithiothreitol (DTT) or tris(2-carboxyethyl)phosphine (TCEP)) to avoid oxidation. However, nitroxide compounds are not compatible with reducing agents because the former are converted from the paramagnetic nitroxide radical −NO^•^ into diamagnetic hydroxylamine −NOH.^27^ Another issue is that in the nitroxide-based method observation of PREs of sufficient magnitude requires high concentrations of the paramagnetic cosolutes (typically ∼10 to 20 mM), which may cause undesired binding to sites on the biomolecule. Furthermore, because the paramagnetic center and the charged group are located on the opposite ends of the nitroxide ring, the resulting PREs can be sensitive to the orientation of the cosolute with respect to the macromolecule, complicating comparison between experiment and calculation in cases where an accurate structure is available.^28^

Here, we present an approach for the measurement of ϕ_EN*S*_ values that largely circumvents the above issues. The new method utilizes two charged Gd chelates (Figure 1): Gd-tetraazacyclododecane-tetraacetate (Gd-DOTA)^30^ and its derivative, Gd-tetraazacyclododecane-bisacetate-bisacetamide (Gd-DOTAM-BA), whose net charges are −1*e* and +1*e*, respectively. Both probes are compatible with reducing agents. Moreover, the seven unpaired electrons in the 4f orbitals of Gd translate into substantially larger measured PREs than what is observed with nitroxide cosolutes at the same concentrations. In this manner, the required concentrations of the Gd chelates are significantly lower, thereby minimizing binding to the biomolecule of interest. Finally, the paramagnetic and charge centers for the Gd chelates are more proximal than for the nitroxide derivatives. Taken together, the Gd-based ϕ_ENS_ method offers improved performance and utility for the measurement of electrostatic potentials of biomolecules over the previously described nitroxide-based approach.

## Methods

### Chemicals

Gd-DOTA (cat# M-147) and Gd-DOTAM-BA (custom order based on the DOTAM-BA chelator [Cat# B-172]) were purchased from Macrocyclics, Inc. (Plano, TX). Gd-HP-DO3A (also known as gadoteridol; Cat# 1287631) was purchased from Sigma-Aldrich (St. Louis, MO). Aminomethyl-PROXYL (Cat# 270180), carboxy-PROXYL (Cat# 253324), and carbamoyl-PROXYL (Cat# C5151) were purchased from Sigma-Aldrich. Other chemicals were also purchased from Sigma-Aldrich unless indicated otherwise.

### Proteins and DNA

^15^N,^13^C-labeled ubiquitin, ^2^H,^15^N G48A Fyn SH3 domain, ^15^N HMGB1 A-box domain, and ^15^N RtoK CAPRIN1 low-complexity domain (C-terminal 103 residues of full-length CAPRIN1 with all 15 Arg in the WT sequence replaced by Lys; in what follows this fragment is referred to as RtoK CAPRIN1) were expressed in *Escherichia coli* and purified as previously described.^31−36^ An ^15^N,^13^C-labeled 15-bp DNA duplex (sequence: 5′-CCAAAGCCATTAGGG-3′) was enzymatically produced and purified as described.^37^

### Gd-Chelate and PROXYL-Stock Solutions

Since powders of Gd-DOTA and Gd-DOTAM-BA are hygroscopic and contain unknown amounts of Na^+^/Cl^–^ ions and water, the material weight used to prepare a solution does not provide an accurate molar concentration. Gd concentrations in stock solutions were measured using the Evans method.^38^ Since Gd governs the overall molar magnetic susceptibility of the Gd chelates, the Gd concentration (in M) can be determined from the experimentally observed magnetic susceptibility shift, Δ (ppm), using the relation
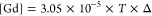
1where *T* is the temperature in K. This equation is obtained from eq 2 of Corsi et al.^38^ together with μ_eff_ = 7.94 for Gd from Table 6 of Peters et al.^39^

As described previously,^20^ concentrations of stock PROXYL compounds can be obtained by reducing the cosolute using ascorbate and then recording fully relaxed one-dimensional ^1^H NMR spectra of samples containing a known concentration of a dimethyl sulfoxide (DMSO) standard. Comparison of integrals of signals from the PROXYL and the DMSO methyl groups provides a facile way of obtaining accurate cosolute concentrations. This approach has been used here for the PROXYL derivatives. In principle, the Evans method can also be used to quantify the PROXYL derivatives if their molar magnetic susceptibility is determined.

### Relaxivities of Paramagnetic Cosolutes Based on Water Measurements

Relaxivities were compared for Gd-DOTA, Gd-DOTAM-BA, carboxy-PROXYL, and aminomethyl-PROXY by recording longitudinal relaxation rates (Γ_1_) of the ^1^H nuclei of water.^23^ Similar experiments can also be performed on samples of the biomolecules under study using ^1^H relaxation of the water solvent so as to accurately quantify the concentrations of cosolutes using standard curves of ^1^H Γ_1_ PRE vs [paramagnetic cosolute], as described below and previously.^23^ The pulse program used for the water relaxivity measurement is given in the SI.

### NMR Experiments to Measure ^1^H Transverse PRE Rates (Γ_2_) for Proteins and DNA

We refer to PREs arising from the addition of paramagnetic cosolute molecules as “solvent PREs” as is commonly done in the literature.^40,41^ The NMR experiments for ubiquitin were performed using 500 μL solutions of 0.3 mM ^15^N,^13^C-labeled protein in a buffer of 20 mM Tris·acetate (pH 7.5) and 5% D_2_O with and without 2.1 mM Gd-DOTA or 3.8 mM Gd-DOTAM-BA. For the reduced and oxidized states of the HMGB1 A-box domain, the samples comprised 500 μL solutions of 0.3 mM ^15^N-labeled protein in a buffer of 10 mM potassium phosphate (pH 7.4), 100 mM NaCl, and 5% D_2_O (plus 5 mM DTT for the reduced state only) with and without 1 mM Gd-DOTA or 3 mM Gd-DOTAM-BA. For ubiquitin and the HMGB1 A-box protein, solvent PRE rates (Γ_2_) were measured at 25 °C via the two time-point approach using heteronuclear two-dimensional (2D) ^1^H spin–echo pulse schemes with a transverse relaxation delay of 10 ms, as previously described.^20,31,42^ For the 15-bp DNA duplex, solvent PREs were measured at 25 °C using 4.9 mM Gd-DOTA or 0.50 mM Gd-DOTAM-BA in 500 μL solutions of 0.3 mM ^15^N,^13^C-labeled DNA in a buffer of 10 mM potassium phosphate (pH 7.4), 100 mM NaCl, and 5% D_2_O, via the two time-point approach using ^1^H–^13^C HSQC-type ^1^H spin–echo pulse schemes with a transverse relaxation delay of 14 ms, as described previously.^43^ PRE Γ_2_ rates for DNA H6/H8 protons and for DNA H2′/H2″/CH_3_ protons were separately measured using ^13^C settings optimized for DNA C6/C8 (134–145 ppm) or C2′/CH_3_ (13–43 ppm) groups and homonuclear ^13^C-J refocusing for C5/C1′/C3′ (75–100 ppm), as described.^43^ H2′/H2″ rather than H1′ protons were chosen for the current study because the H2′/H2″ resonances are more distant from the water ^1^H resonance; proximity to the water line can cause baseline distortions and, therefore, may adversely affect Γ_2_ measurements. A Bruker AVANCE III spectrometer with a QCI cryogenic probe operating at a ^1^H frequency of 600 MHz was used for these experiments. PREs, recorded using a Bruker AVANCE NEO 1 GHz spectrometer with a TCI cryogenic probe, were measured on 500 μL solutions of 0.14 mM ^2^H,^15^N-labeled G48A Fyn SH3 domain in 20 mM sodium phosphate (pH 6.0), 5% D_2_O at 10 °C, both with and without one of a number of cosolutes. These included 0.99 mM Gd-DOTA, 0.14 mM Gd-DOTAM-BA, 30 mM carboxy-PROXYL, and 5 mM aminomethyl-PROXYL. Similar experiments were recorded on 0.3 mM samples of ^15^N-labeled RtoK CAPRIN1 dissolved in 25 mM MES (pH 5.5), 5% D_2_O. In this case, the cosolutes were 0.49 mM Gd-DOTA, 1.30 mM Gd-DOTAM-BA, 5 mM carboxy-PROXYL, or 5 mM aminomethyl-PROXYL, 25 °C. A gradient-enhanced pulse sequence was used to measure amide proton transverse relaxation rates for the G48A Fyn and RtoK CAPRIN1 samples, as described in the literature.^44^

### Determination of ϕ_ENS_ Potentials

Effective near-surface electrostatic potentials, ϕ_ENS_, for individual ^1^H nuclei in each of the systems studied were determined from solvent PRE rates^20^
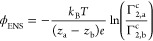
2where Γ_2,a_^c^ and Γ_2,b_^c^ are the concentration-normalized transverse PRE rates arising from the added paramagnetic cosolutes with charges *z*_a_ and *z*_b_, respectively; *k*_B_ is Boltzmann’s constant; *T* is the absolute temperature; *e* is the elementary charge. The concentration-based normalization is given by
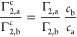
3where Γ_2,a_ and Γ_2,b_ rates are experimentally determined PRE rates and *c*_a_ and *c*_b_ are the concentrations of the paramagnetic cosolutes, established as described above. The uncertainty in each ϕ_ENS_ value was estimated through error propagation^45^ using
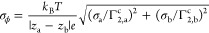
4where σ_a_ and σ_b_ are the uncertainties in Γ_2,a_^c^ and Γ_2,b_^c^, respectively.

### Poisson–Boltzmann-Based Electrostatic Potentials

Electrostatic potentials at grid points in a 3D lattice space containing the biomolecule of interest were computed by solving the Poisson–Boltzmann equation using APBS (version 3.0)^14^ and DelPhi (version 8.6)^16^ software packages. Atomic coordinates, point charges, and radii in the PQR format were generated from PDB-format structures using the PDB2PQR program^46,47^ and the AMBER force-field parameter set.^48^ The dimensions of the 3D lattice space were 128 Å × 128 Å × 128 Å for ubiquitin and the Fyn SH3 domain and 160 Å × 160 Å × 160 Å for the 15-bp DNA duplex, and the grid interval was 0.5 Å in each dimension. Examples of the input parameter settings for APBS and DelPhi are provided in the Supporting Information (SI). The output electrostatic potential files (in “dx” format for APBS and in “cube” format for DelPhi) were used to predict effective near-surface electrostatic potentials ϕ_ENS_ (see below).

### Poisson–Boltzmann Theory-Based Prediction of ϕ_ENS_ Potentials

For each ^1^H nucleus, Poisson–Boltzmann theory-based near surface electrostatic potentials, ϕ_ENS_^PB^, were calculated as follows
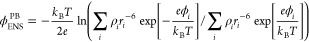
5where *i* represents an index of a grid point; ϕ_*i*_ is the electrostatic potential at grid point *i* (obtained from an APBS “dx” format output or a DelPhi “cube” format output); *r*_*i*_ is the distance from the ^1^H nucleus to the grid point *i*; *ρ*_*i*_ is a factor that represents the accessibility of grid point *i* and is either 1 (accessible) or 0 (inaccessible). A value of 0 was assigned to *ρ*_*i*_ when *d*_min_ < *r*_VDW_ + *r*_pc_, where *d*_min_ is the distance from grid point *i* to the closest atom in the macromolecule with van der Waals radius *r*_VDW_ and *r*_pc_ is the effective radius that defines the accessibility of the PROXYL paramagnetic center (Figure S1). Using the procedures described previously,^20^ the value of *r*_pc_ was empirically optimized to be 3.5 Å (Figure S2). To avoid arithmetic overflow, the exponential terms were not evaluated for grid points with *ρ*_*i*_ = 0. A package (named “PBENS”) containing the MATLAB scripts and the input files for these calculations is available on a GitHub web page (https://github.com/IwaharaLab/PBENS).

## Results

### Experimental Systems under Consideration

**Figure 2:**
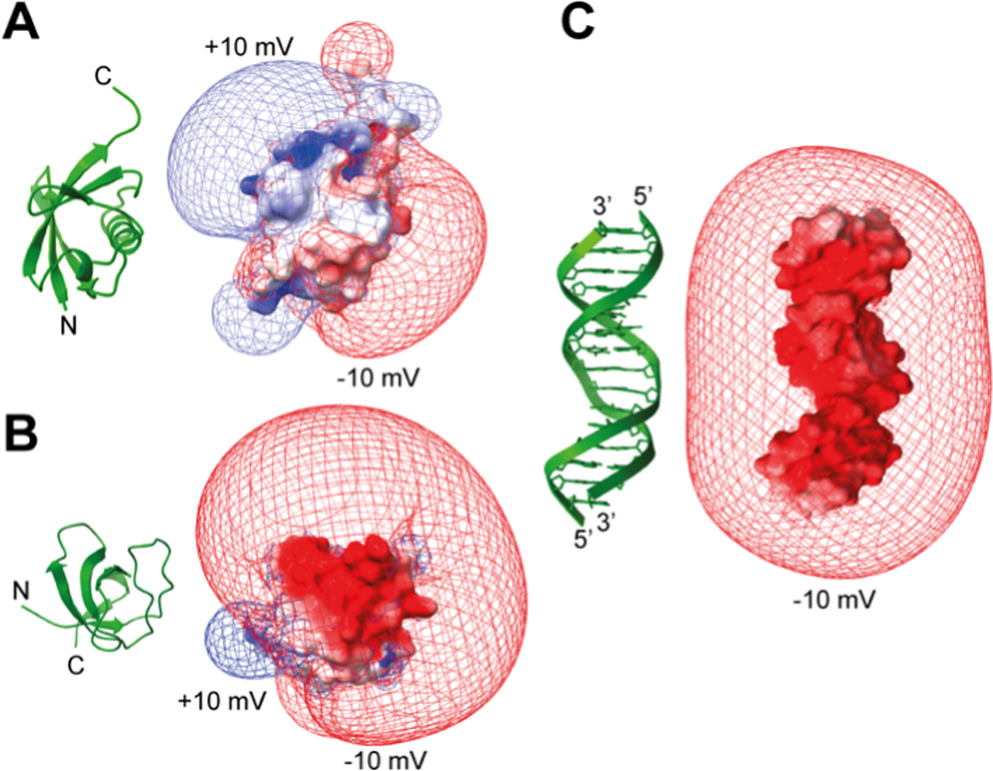
Isopotential maps at ±10 mV for the electrostatic potentials of (A) ubiquitin, (B) G48A Fyn SH3, and (C) 15-bp DNA, drawn with ChimeraX.^49^ For these maps, the APBS program^14^ was used to compute electrostatic potentials at grid points in 3D space by numerically solving the nonlinear Poisson–Boltzmann equation.

To test the Gd-based approach for the measurement of effective near-surface electrostatic potentials, ϕ_ENS_, we have used four biomolecules with different net charges at physiological pH: ubiquitin (76 residues; 0*e*), the G48A Fyn SH3 domain (60 residues; −6.5*e*), the CAPRIN1 low-complexity domain (103 residues, where all Arg are replaced by Lys; +13*e*; referred to as RtoK CAPRIN1 in what follows), and a DNA duplex (15 base pairs; −28*e*). Structures of ubiquitin, G48A Fyn SH3, and the DNA construct studied are available (Figure 2)^50−52^ so that comparisons can be made between measured ϕ_ENS_ values and those obtained via structure-based predictions. In contrast, the RtoK CAPRIN1 low-complexity domain is intrinsically disordered. For ubiquitin, the 15-bp DNA, and RtoK CAPRIN1, ϕ_ENS_ potentials measured with nitroxide cosolutes have been extensively analyzed in previous studies.^20,22,23,28,31,43,53,54^ Thus, these biomolecules provide an excellent reference set for comparison of ϕ_ENS_ values measured using the various types of paramagnetic compounds.

### Relaxivity of the Paramagnetic Cosolutes

**Figure 3:**
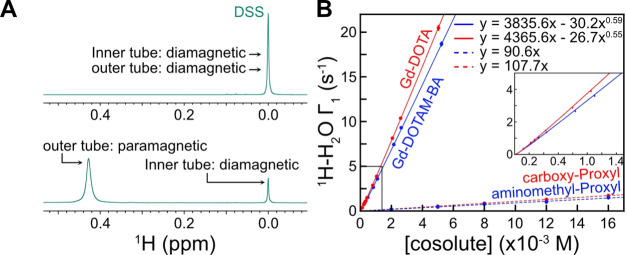
Quantification of the concentrations of the paramagnetic cosolutes and measurements of their relaxivities. (A) Determination of Gd-chelate concentrations by the Evans method using coaxial NMR tubes for a high-field NMR spectrometer.^38^ Because Gd governs the overall molar magnetic susceptibility of the Gd chelates, the Gd concentration can be determined from the experimentally observed magnetic susceptibility shift Δ in ppm using eq 1. Concentrations of the nitroxide-based cosolutes are obtained as described previously.^20^ (B) ^1^H relaxivity data for the Gd chelates and the PROXYL derivatives, focusing on longitudinal relaxation of water ^1^H spins as a function of the concentration of paramagnetic cosolutes. Data were measured at a ^1^H frequency of 500 MHz using a buffer of 25 mM MES (pH 5.5) and 1 mM DSS at 25 °C using a saturation-recovery method described by Toyama et al.^23^ The second term of the regression was introduced to account for slight deviation from linearity. Water ^1^H relaxivity data allow for calibration of the paramagnetic cosolute concentration in biomolecular samples in the same buffer.

Relaxivity, a parameter commonly associated with magnetic resonance imaging contrast agents,^55^ is defined as the solvent PRE rate per millimolar concentration of paramagnetic cosolute. This parameter provides a useful gauge for comparing how effective different paramagnetic cosolutes are in relaxing proximal nuclear spins. As a first step toward measuring the relaxivity of the Gd chelates used in the present study, we quantified their concentrations using the Evans method.^38^ An example of Gd quantification by the Evans method is shown in Figure 3A. Here, a pair of coaxial tubes is used, both containing a test compound dissolved in buffer, with the paramagnetic probe of interest added to the outer tube. In the example of Figure 3A, sodium 2,2-dimethyl-2-silapentane-5-sulfonate (DSS) is used as a test compound, and the shift difference of the methyl ^1^H signals derived from the DSS in the inner and outer tubes can be directly translated into the absolute concentration of the paramagnetic cosolute using eq 1. After establishing the concentrations of Gd-based cosolutes in this manner and the nitroxide cosolutes as described in Materials and Methods, we measured their relaxivities using the ^1^H nuclei of water (Figure 3B). Here, ^1^H PRE longitudinal relaxation rates, Γ_1_ (difference in relaxation in the presence and absence of cosolute), are plotted as a function of the concentration of the paramagnetic cosolute. As shown in Figure 3B, the relaxivities of the Gd chelates are significantly larger than those of the corresponding nitroxide compounds, by 24–34 fold. That is, the Gd chelates can generate substantially larger solvent PREs than the nitroxide compounds at the same concentration, an important advantage in studies of biomolecules, in particular, that may possess binding sites for the cosolutes.^56^ It is worth noting that standard plots of water relaxivity vs concentration profiles of the sort illustrated in Figure 3 are useful for the accurate estimation of the concentration of paramagnetic cosolutes in samples of the biomolecule of interest.^23^ We found that the water relaxivity depends slightly (but statistically significantly) on the magnetic field (11.7–23.4 T), the buffer, and the Gd concentration range. Therefore, standard plots calibrating the Gd concentrations, as in Figure 3B, should be obtained *using solutions with the same buffer and under the same experimental conditions as for the actual biomolecular sample*. Knowledge of the concentrations of paramagnetic compounds used is critical for obtaining accurate ϕ_ENS_ values (eq 3).

### Solvent PREs Arising from the Gd Chelates

**Figure 4:**
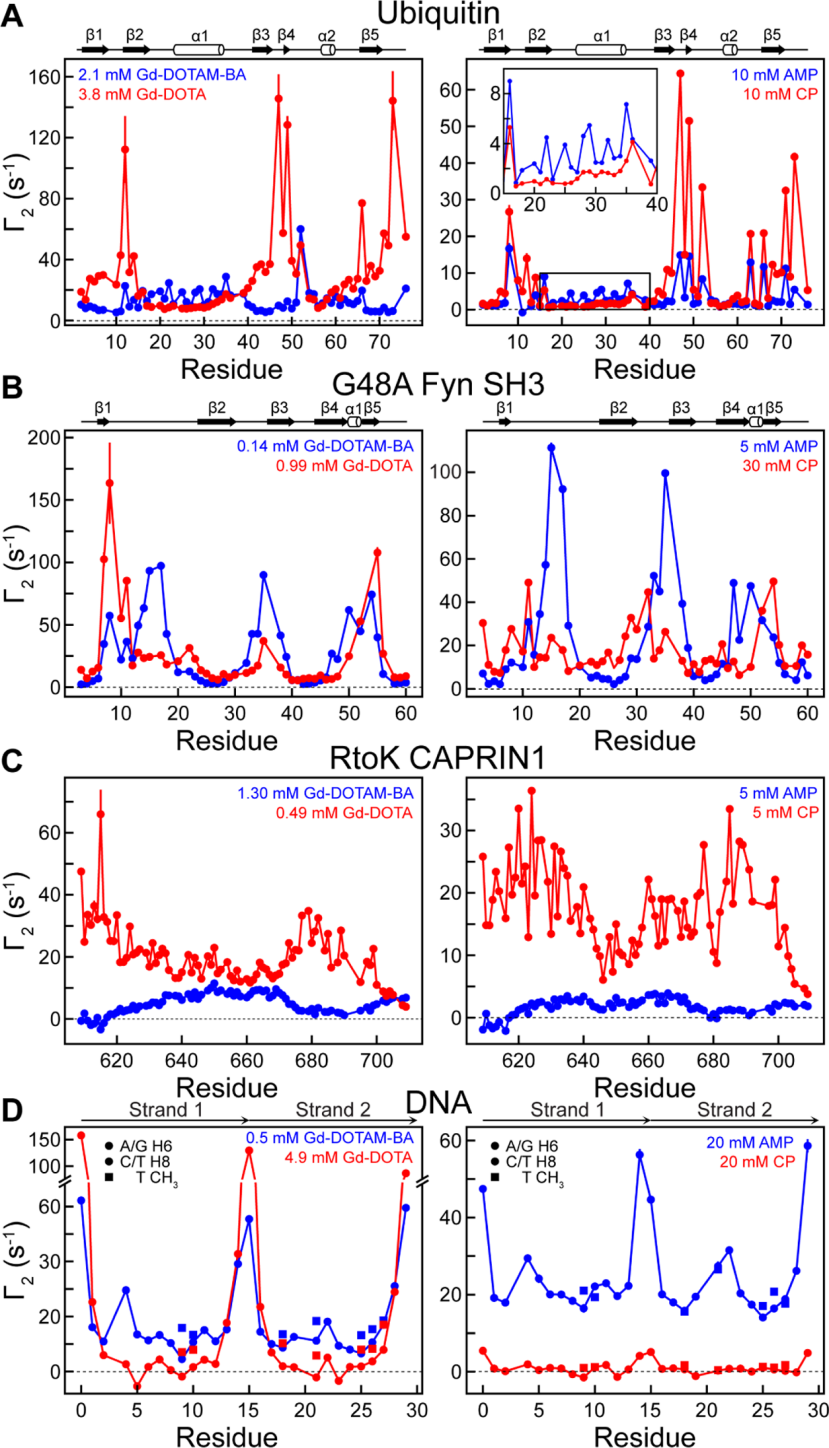
Transverse ^1^H PRE rates measured with Gd-DOTA (−1*e*) and Gd-DOTAM-BA (+1*e*) (left-hand side) or with carboxy-PROXYL (CP; −1*e*) and aminomethyl-PROXYL (AMP; +1*e*) (right-hand side) cosolute pairs. The concentrations of the paramagnetic cosolutes used are indicated. (A) Backbone ^1^H_N_ Γ_2_ data for 0.3 mM ^15^N-labeled ubiquitin in a buffer of 20 mM Tris·acetate (pH 7.5) and 5% D_2_O at 25 °C. (B) Backbone ^1^H_N_ Γ_2_ data for 0.14 mM ^2^H,^15^N-labeled G48A Fyn SH3 in 20 mM sodium phosphate (pH 6.0), 5% D_2_O at 10 °C. (C) Backbone ^1^H_N_ Γ_2_ data for ^15^N-labeled RtoK CAPRIN1 dissolved in 25 mM MES (pH 5.5), 5% D_2_O. (D) Γ_2_ data for 0.3 mM ^13^C,^15^N-labeled 15-bp DNA in a buffer of 10 mM potassium phosphate (pH 7.4), 100 mM NaCl, and 5% D_2_O at 25 °C. The negative Γ_2_ values observed for H6/H8 of G5, T21, and G23 could be due to pulse imperfections^44^ as their C6/C8 resonances were near the edge of the bandwidth of the ^13^C Q3 selective pulses used. Secondary structure diagrams are shown above the profiles for ubiquitin and G48A Fyn SH3.

We have measured solvent PREs for ^1^H nuclei of ^15^N,^13^C-labeled ubiquitin, ^2^H,^15^N-labeled G48A Fyn SH3, ^15^N-labeled RtoK CAPRIN1, and ^15^N,^13^C-labeled 15pb DNA, using heteronuclear NMR experiments, as described previously,^40,43,44^ in the presence and in the absence of the paramagnetic cosolutes (Gd-DOTA, Gd-DOTAM-BA, aminomethyl-PROXYL, carboxy-PROXYL). Some examples of spectra are shown in Figure S3. Figure 4 plots ^1^H transverse PRE rates, Γ_2_, measured for ubiquitin (A), G48A Fyn SH3 (B), RtoK CAPRIN1 (C), and 15-bp DNA (D) using the concentrations of the various cosolutes indicated in the figure. The PRE rates for the proteins were measured for backbone ^1^H_N_ nuclei, whereas those for the 15-bp DNA are for H2′, H2″, H6, H8, and methyl protons. The relaxation trends, especially for residues with large PRE values, were qualitatively similar between the Gd- and PROXYL-based datasets; however, as expected, the concentrations of the Gd chelates required to generate similar PRE values were, on average, significantly lower than for the PROXYL derivatives.

The large relaxivities of the Gd chelates are particularly useful in studies of highly charged systems. In a previous report, involving the 15-bp DNA duplex, only very small PRE values could be measured when using the carboxy-PROXYL (−1*e*) derivative, even at a relatively high concentration of 20 mM, that reflects the electrostatic repulsion between the two negatively charged molecules.^43^ As a result, the neutral carbamoyl-PROXYL cosolute was substituted for the carboxy-PROXYL compound for the determination of ϕ_ENS_ potentials although the use of neutral paramagnetic cosolutes can compromise the accuracy of the measured electrostatic potentials, as discussed previously^54^ and below. Notably, using a relatively low Gd-DOTA concentration (−1*e*, 4.9 mM), it was possible to measure sufficiently large solvent PREs so as to enable the determination of robust ϕ_ENS_ potentials for the 15-bp DNA fragment (Figure 4D).

The comparatively larger PREs measured when using the Gd compounds vs the nitroxide cosolutes can be appreciated by considering the expression for the PRE rate^20,57^
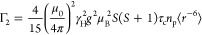
6where it is assumed that the biomolecule in question tumbles slowly in solution so that the spectral density function evaluated at zero frequency is much larger than the value at ω_*H*_ (^1^H Larmor frequency in rad s^–1^). In eq 6, μ_0_ is the vacuum permeability, γ_H_ is the ^1^H nuclear gyromagnetic ratio, *g* is the electron *g*-factor, μ_B_ is the Bohr magneton, *S* is the electron spin quantum number for the paramagnetic cosolute (7/2 for the Gd chelates and 1/2 for the nitroxide compounds), τ_c_ is the correlation time for the magnetic dipole–dipole interaction between the ^1^H spin attached to the macromolecule and the unpaired electron of the cosolute, *r* is the distance between the unpaired electron and the ^1^H nucleus, and < > denotes an ensemble average. The parameter *n*_p_ is the number of paramagnetic cosolute molecules in a unit volume (i.e., 1 m^3^), where *n*_p_ = 1000*N*_A_*c*_p_, with *N*_A_ being Avogadro’s number and *c*_p_ being the cosolute concentration in mol/L units. The larger PREs for Gd result, in large part, from the fact that the factor *S*(*S* + 1) is 21 times larger than for nitroxides. Interestingly, our experimental data show that the ratio of PREs for the Gd vs nitroxide cosolutes can be different from the factor of 21, varying from ∼3 (e.g., Gd-DOTAM-BA vs aminomethyl-PROXYL for H_N_ of G47 in ubiquitin) to as large as 60 (Gd-DOTA vs carboxy-PROXYL for H_N_ of D39 in ubiquitin). This difference must arise from discrepancies between the correlation time τ_c_ and/or the <*r*^–6^> term in eq 6 for different cosolutes. The correlation times, τ_c_, for solvent PREs derived from nitroxides, for example, do not depend on the electron *T*_1_ that is slow (∼1 μs)^58^ but rather on the diffusion of the nitroxide.^57^ In contrast, the electron relaxation times for the Gd chelates are much faster and, therefore, likely to contribute to τ_c_.^59^ Additionally, the shortest distance between the observed ^1^H nucleus and the paramagnetic center could be different between the two types of cosolutes. Nonetheless, owing to the large electron spin quantum number, it is clear that Gd chelates will result in substantially larger solvent PREs than their nitroxide counterparts, for similar concentrations of compounds. This feature is practically useful. Small Γ_2_ rates (e.g., <1 s^–1^) are difficult to precisely measure because the available range of ^1^H spin–echo lengths can be limited by rapid ^1^H transverse relaxation even for diamagnetic samples.

### ϕ_ENS_ Potentials Measured Using Gd and PROXYL Derivatives

**Figure 5:**
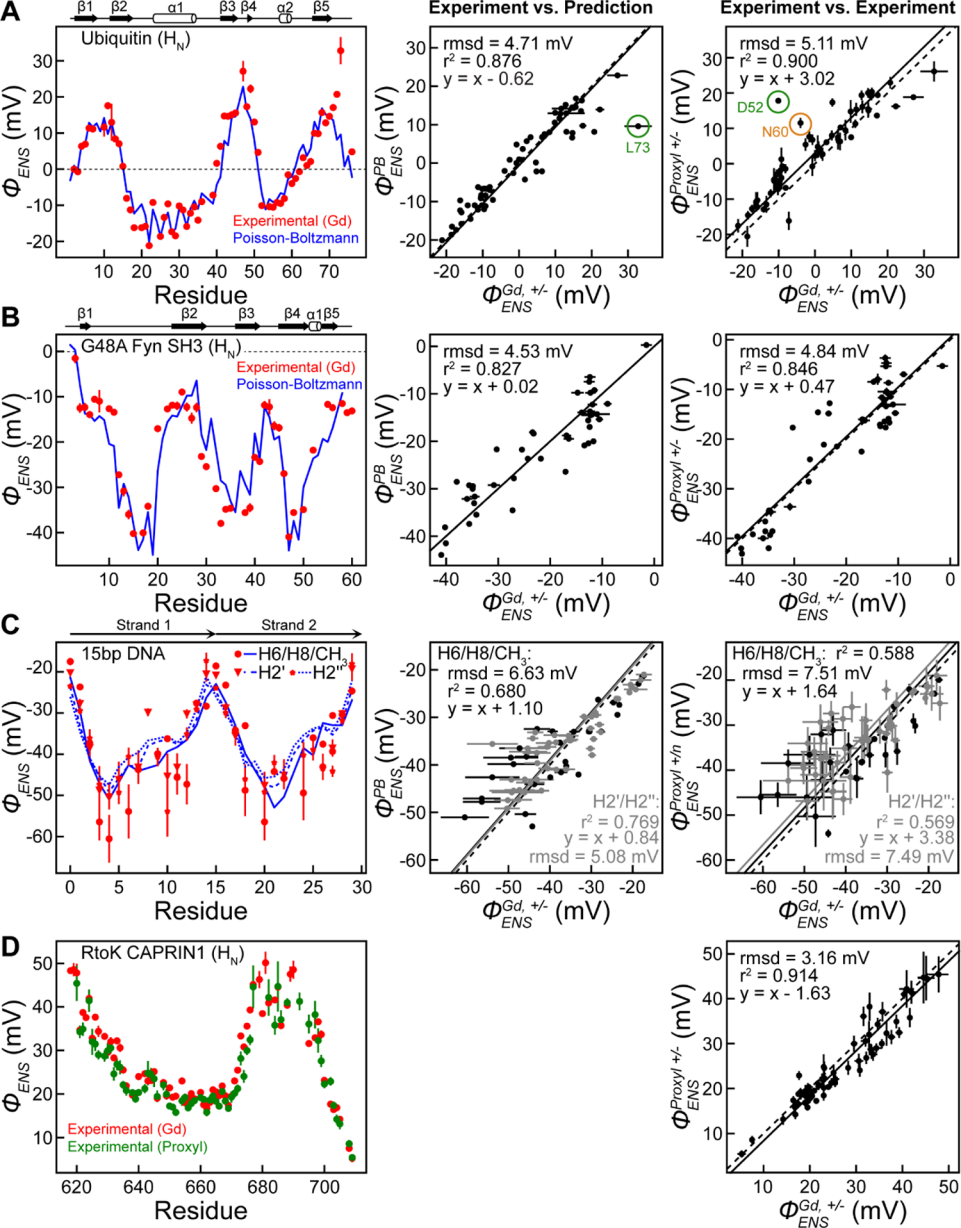
Comparison of measured and calculated electrostatic potentials for a range of biomolecular systems. ϕ_ENS_ potentials measured using Gd-DOTA (−1*e*) and Gd-DOTAM-BA (+1*e*) are shown in red for (A) ubiquitin, (B) G48A Fyn SH3, (C) 15-bp DNA, and (D) RtoK CAPRIN1. ϕ_ENS_ values were determined from the solvent PRE data shown in Figure 4 using eq 2. In (A–C), the measured ϕ_ENS_ potentials are compared with potentials obtained via Poisson–Boltzmann calculations (ϕ_ENS_^PB^), which are shown in blue, and the middle panels show correlations between the experimental (ϕ_ENS_^Gd^) and predicted (ϕ_ENS_^PB^) potentials. The right panels show correlations between the experimental potentials measured with the Gd chelates and those measured with PROXYL derivatives. In (A, B, D), ϕ_ENS_^Proxyl^ values were measured using amino-methyl-PROXYL (+1*e*) and carboxy-PROXYL (−1*e*) cosolutes, while in (C), ϕ_ENS_^Proxyl+/*n*^ values were measured using aminomethyl-PROXYL (+1*e*) and carbamoyl-PROXYL (neutral) cosolut*e*s. In (D), ϕ_ENS_ potentials m*e*asured using carboxy-PROXYL (−1*e*) and aminomethyl-PROXYL (+1*e*) are shown in green, and a correlation plot for the two experimental datasets (Gd- and PROXYL-based) is shown. In each correlation plot, the dotted line is the diagonal (i.e., *y* = *x*), whereas the solid line, *y* = *x* + *a*, is based on linear regression. Outlier ϕ_ENS_ values (residues D52, N60, L73) are circled in (A) and discussed in the text. D52 and N60 were excluded in the calculation of RMSD between ϕ_ENS_^Gd^ and ϕ_ENS_^Proxyl^. See text and SI for discussion. Error bars were obtained using eq 4. In (C), errors in ϕ_ENS_ potentials for CH_3_ groups are far smaller than those for other DNA ^1^H nuclei.

Having measured solvent PRE rates for the four biomolecular systems under investigation, we next calculated ϕ_ENS_ potentials as a function of residue from the ratio of Γ_2_ values measured using positive and negative cosolutes (eq 2). As accurate ϕ_ENS_ values are predicated on relaxation rates obtained with known concentrations of cosolutes (eq 3), we first quantified concentrations based on measurements similar to those described in the context of Figure 3. Figure 5A (left) displays the ϕ_ENS_ potentials of individual residues for ubiquitin (red), from measurements using Gd-DOTA (−1*e*) and Gd-DOTAM-BA (+1*e*). By means of comparison, we have also calculated ϕ_ENS_ potentials using the X-ray structure of ubiquitin and eq 5, as described in detail previously,^20^ based on Poisson–Boltzmann theory (Figure 5A, left, blue curve; Figure 5A, center). Similar predictions obtained with the DelPhi package were consistent with those using the APBS software illustrated here (Figure S4). Despite the different chemical structures of the Gd- and PROXYL-based derivatives (Figure 1), ϕ_ENS_ potentials determined using the Gd-DOTA and Gd-DOTAM-BA pair agreed well with those from carboxy-PROXYL (−1*e*) and aminomethyl-PROXYL (+1*e*) derivatives (RMSD, 5.1 mV, excluding D52 and N60; Figure 5A, right). Thus, in addition to validating our measurements using computation, it is possible to establish their robustness in a structure-independent manner based on NMR measurements exclusively.

Notably, a few residues of ubiquitin exhibited significant differences between ϕ_ENS_ potentials, as measured using the two sets of cosolutes (Figure 5A, right). For the backbone ^1^H_N_ groups of D52 and N60, particularly large discrepancies between PROXYL-based and predicted ϕ_ENS_ values were observed previously,^20^ which were subsequently explained in a later computational study^28^ in terms of preferential orientations of the PROXYL paramagnetic probes with respect to the protein, coupled with the separation of the positions of the charge and the paramagnetic center in these compounds. The discrepancies for these two residues disappeared when the ϕ_ENS_ potentials were measured using the Gd chelates (Figure 5A, left). In principle, preferential orientations of the cosolutes would have less impact on observed ϕ_ENS_ values if both the paramagnetic center and the net charge center were close to the molecular center. This can explain the improved agreement between the experimental ϕ_ENS_ values and the predictions for D52 and N60 because the paramagnetic center and the charged groups are proximal in the Gd chelates, whereas they are on opposite sides of the nitroxide ring in the PROXYL derivatives (Figure 1).

The ϕ_ENS_ potentials measured for ubiquitin H_N_ atoms using the Gd chelates generally agreed well with the predictions from the crystal structure (Figure 5A, middle). However, a significant difference between the experimental and predicted ϕ_ENS_ values was observed for L73 in the C-terminal tail. As explained in the SI (see also Figure S5), this discrepancy appears to be largely due to differences between the dynamic conformational ensemble of the C-terminal tail in solution and the static structure of the tail immobilized by intermolecular packing in the crystal structure.^60^ The RMSD between the experimental and predicted ϕ_ENS_ potentials for H_N_ atoms in ubiquitin was significantly smaller with Gd-DOTA/Gd-DOTAM-BA than with the PROXYL derivatives (Table 1). The corresponding data for H_α_ and methyl protons of ubiquitin are shown in Figure S6, with significantly smaller RMSDs for ϕ_ENS_ potentials measured using the Gd chelates (4.8 mV for H_α_; 3.2 mV for CH_3_) than the corresponding values based on the PROXYL derivatives (7.0 mV for H_α_; 5.6 mV for CH_3_).^31^

**Table 1:** RMSDs between the Experimental ϕ_ENS_ Potentials and the Poisson–Boltzmann Theory-Based Predictions^a^

		paramagnetic cosolutes	
macromolecule	^1^H type	Gd-DOTA (−1*e*) Gd-DOTAM-BA (+1*e*)	carboxy-PROXYL (−1*e*) aminomethyl-PROXYL (+1*e*)
ubiquitin	H_N_	4.7 (3.7)^b^	6.9 (5.4)^b^^,^^c^
	H_α_	4.8	7.0^d^
	CH_3_	3.2	5.6^d^
G48A Fyn SH3	H_N_	4.5	5.9
15-bp DNA	H_C_^e^	5.7^f^	n.d.^g^

a The values are in units of mV.

b The values in the parentheses include only regions of stable secondary structure.

c From ref (20).

d From ref (31).

e DNA H2′, H2″, H6, H8, and T CH_3_ protons.

f Averaged over all DNA protons.

g No data. Solvent PRE rates with carboxy-PROXYL (−1*e*) were too small to determine ϕ_ENS_ potentials.

Figure 5B shows similar ϕ_ENS_ data as presented in Figure 5A but for the G48A Fyn SH3 domain, using the Gd-DOTA and Gd-DOTAM-BA pair. Unlike ubiquitin, which is neutral, this protein is negatively charged (see Figure 2), and, not surprisingly, therefore, the measured ϕ_ENS_ potentials were largely negative. The agreement with the calculated values (APBS software) is significantly better for the Gd chelates (4.5 mV) relative to the potentials from the PROXYL derivatives (RMSD of 5.9 mV) (Table 1).

We have applied the Gd-based method to measure electrostatic potentials of DNA as well. Owing to the large solvent PREs arising from Gd, we were able to use the negatively charged cosolute, along with its positively charged counterpart, to determine ϕ_ENS_ potentials. Figure 5C plots the experimentally derived ϕ_ENS_ values for H2′/H2″, H6, H8, and methyl H atoms of the 15-bp DNA (red) along with predictions using APBS software (blue), both as a function of residue (left) and in the form of a correlation plot (middle). To calculate the predicted values, the DNA portion from the crystal structure of the Antp homeodomain-DNA complex (PDB 9ANT)^52^ was used since the structure of this DNA in the free state was unavailable. The RMSD between the experimental and predicted ϕ_ENS_ potentials is 6.6 mV, larger than obtained for ubiquitin and G48A Fyn SH3, perhaps due to structural differences between the protein-bound and free DNA molecules^61^ or, additionally, potentially reflecting the known problems using Poisson–Boltzmann theory for highly charged systems.^62,63^

Finally, as an example of an application to an intrinsically disordered protein, we have measured ϕ_ENS_ potentials for RtoK CAPRIN1 using both Gd- and PROXYL derivatives (Figure 5D). Similar trends in the profiles were observed, along with a reasonable agreement between the ϕ_ENS_ values obtained with the different cosolutes (RMSD = 3.16 mV). The fact that the completely different pairs of charged paramagnetic cosolutes gave consistent ϕ_ENS_ potentials assures that the ϕ_ENS_ method can provide accurate electrostatic information even for intrinsically disordered proteins (IDPs). This is important because structure-based assessment of experimental ϕ_ENS_ data is difficult for IDPs and IDRs in general.

### Erroneous ϕ_ENS_ Values Based on Neutral Paramagnetic Cosolutes

**Figure 6:**
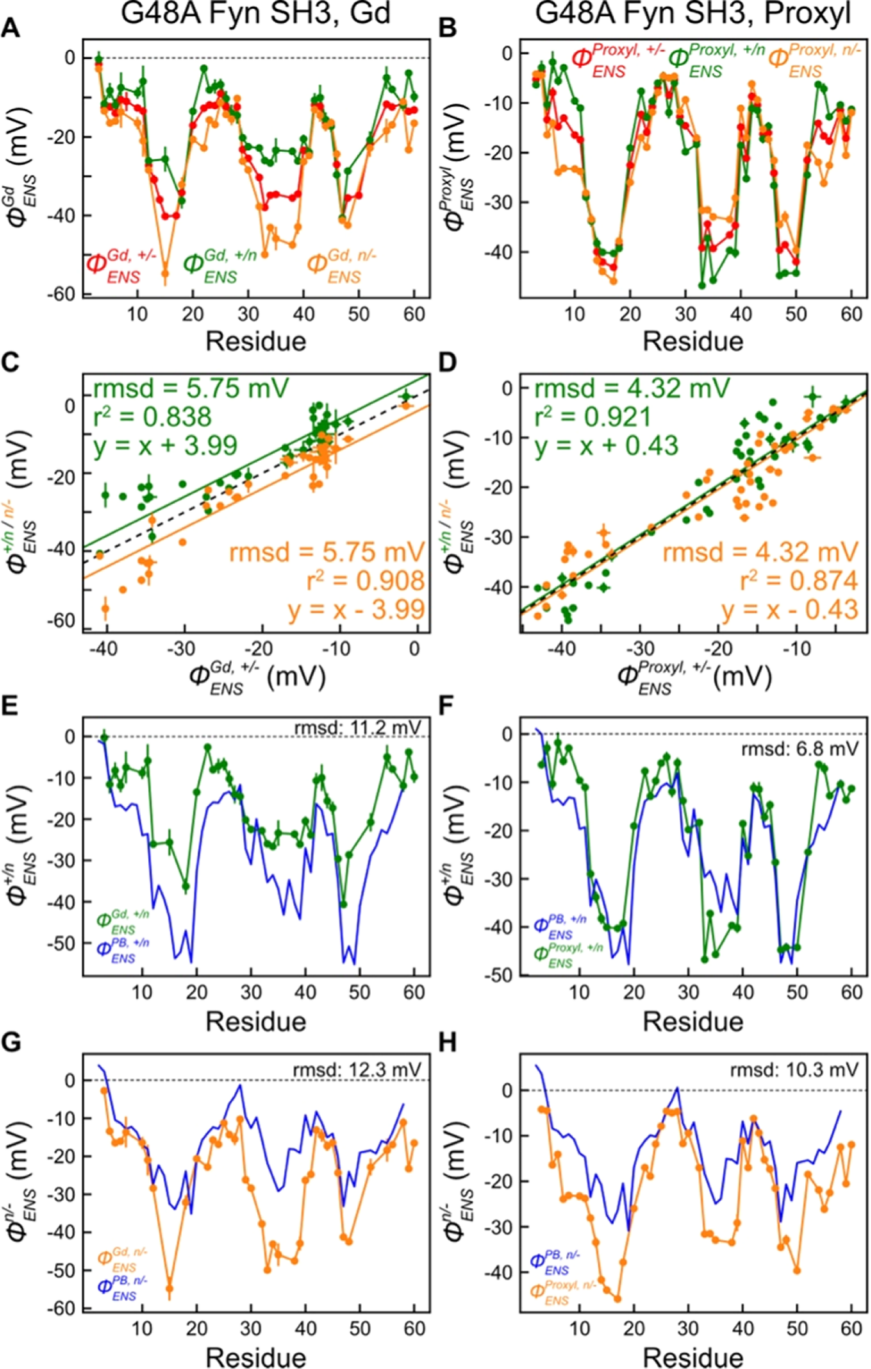
Decreased accuracy of ϕ_ENS_ potentials when measured using neutral paramagnetic cosolutes. Shown are experimental ϕ_ENS_ potentials and Poisson–Boltzmann equation-based predictions (blue in (E–H)) for G48A Fyn SH3. (A, C, E, G) ϕ_ENS_ potentials measured with the +/–, +/*n*, and *n*/– pairs of Gd cosolutes (+, Gd-DOTAM-BA [+1*e*]; *n*, Gd-HP-DO3A [neutral]; −, Gd-DOTA [−1*e*]) along with theoretical predictions (blue). (B, D, F, H) ϕ_ENS_ potentials measured with the +/–, +/*n*, and *n*/– pairs of PROXYL cosolutes (+, aminomethyl-PROXYL [+1*e*]; n, carbamoyl-PROXYL [neutral]; −, carboxy-PROXYL [−1*e*]) along with theoretical predictions (blue). Similar comparisons of electrostatic potentials generated using the three combinations of cosolutes for RtoK CAPRIN1 are shown in Figure S7.

A previous study quantified the electrostatic properties of WT CAPRIN1 during its ATP-induced phase separation trajectory (0 mM NaCl), using cationic, anionic, and neutral PROXYL cosolutes.^23^ Notably, excellent agreement between ϕ_ENS_ potentials was obtained when calculated using any pair of the paramagnetic compounds. Subsequently, when the measurements were repeated in the presence of NaCl, the level of agreement decreased, especially for high concentrations of salt (>500 mM).^54^ Based on studies using either PROXYL or TEMPO derivatives and using either CAPRIN1 or ubiquitin as test protein systems, it was concluded that electrostatic potentials obtained using the +/– pair of radicals were less error prone than for the neutral (carbamoyl-PROXYL)/– and +/neutral combinations. We were interested to examine whether a similar situation might occur for the Gd-based cosolutes as well, and, therefore, measured additional data using the Gd-HP-DO3A cosolute (Figure 1, also known as gadoteridol), a neutral analogue of Gd-DOTA and Gd-DOTAM-BA. Figure 6A,B plots ϕ_ENS_ values for G48A Fyn SH3 calculated from various combinations of Γ_2_ rates recorded using the charged/neutral Gd and PROXYL cosolutes, respectively. Clear differences are observed for potentials obtained using different combinations of measured PRE rates (Figure 6A–D), in particular for the Gd compounds. It is clear that the previous anomalies observed for the PROXYL- and TEMPO-neutral cosolutes are recapitulated here for their Gd-based counterparts. This is further illustrated in Figure 6E–H where the agreement between predicted and measured ϕ_ENS_ values is shown, in general, to be poor when PRE rates from the neutral cosolute are included and certainly worse than when comparing potentials calculated from the +/– pair (Figure 5B).

We compared ϕ_ENS_ values obtained for RtoK CAPRIN1 and again noted that unlike the case for the WT protein, the potentials for the +/neutral and neutral/– pairs were not consistent with values measured with the +/– cosolutes (Figure S7). Issues associated with the neutral paramagnetic cosolute can also be observed in Figure 5C (right) for the 15-bp DNA, where there is relatively poor agreement between ϕ_ENS_^Gd+/–^ and ϕ_ENS_^Proxyl+/*n*^.

Why does using the neutral paramagnetic cosolute lead to large errors? One possibility is related to the assumption that nonelectrostatic contributions, including hydrophobic interactions, are canceled in calculations of ϕ_ENS_ due to the analogous chemical structures of the paramagnetic cosolute pairs that are used in the measurements.^20,28^ Based on eq 8 of Chen et al.,^28^ the apparent ϕ_ENS_ potentials can be expressed as follows
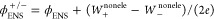
7
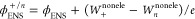
8
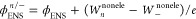
9where *W*^nonele^ is the nonelectrostatic term in the potential of mean force within the effective near-surface (ENS) zone and is defined for each type of paramagnetic cosolute. If the *W*^nonele^ terms are virtually equal due to the analogous chemical structures, then the nonelectrostatic contributions are canceled, leading to ϕ_ENS_^+/–^ = ϕ_ENS_^+/*n*^ = ϕ_ENS_^*n*/–^ = ϕ_ENS_. Alternatively, if the *W*^nonele^ terms are different for each cosolute, then the residual nonelectrostatic terms may be significant, leading to the observed discrepancies. Good agreement between the experimental ϕ_ENS_^+/–^ potentials and the Poisson–Boltzmann theory-based predictions suggests that the residual nonelectrostatic term is generally small compared to the true ϕ_ENS_. However, even the smallest RMSD in Table 1 is considerably larger than typical uncertainties in measured potentials (i.e., 3.2 vs ∼0.3 mV for ubiquitin CH_3_ groups). The residual nonelectrostatic term may be a major source of error in measured ϕ_ENS_^+/–^ potentials. Importantly, since the denominator of the term is *e* in eqs 8 and 9 rather than 2*e* in eq 7, the residual nonelectrostatic term in the ϕ_ENS_^+/*n*^ and ϕ_ENS_^*n*/–^ potentials could be twice as large as for ϕ_ENS_^+/–^ so that ϕ_ENS_^+/*n*^ and ϕ_ENS_^*n*/–^ are less accurate than ϕ_ENS_^+/–^. Further, according to eq 4, the use of a neutral paramagnetic cosolute also makes ϕ_ENS_ measurements less precise. Assuming an identical relative error in PREs (i.e., σ/Γ_2_), the errors in ϕ_ENS_ potentials determined using solvent PRE data with the +/neutral or neutral/– pairs of paramagnetic cosolutes (|*z*_a_ – *z*_b_| = 1) are expected to be twice as large as errors in ϕ_ENS_ values determined with the +/– pair (|*z*_a_ – *z*_b_| = 2). Thus, the use of a neutral paramagnetic cosolute adversely impacts both accuracy and precision of ϕ_ENS_ measurements.

### Gd Chelates Are Compatible with DTT

**Figure 7:**
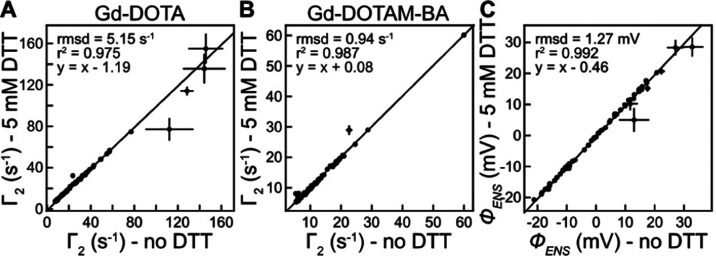
5 mM DTT does not affect electrostatic potential measurements using the Gd chelates. (A, B) Linear correlation plots of measured solvent PRE rates Γ_2_ for backbone ^1^H_N_ nuclei of ubiquitin in the presence and absence of 5 mM DTT. (C) Correlation plot of ϕ_ENS_ potentials measured for ubiquitin in the presence and absence of 5 mM DTT using the PRE rates in (A, B).

DTT is a strong reducing agent used in many biochemical and biophysical experiments to avoid oxidation of protein cysteine side-chain thiol groups. In some previous studies, solvent PREs arising from the addition of neutral Gd chelates (Gd-DTPA-BMA or Gd-HP-DO3A) were measured for protein samples in the presence of 1–5 mM DTT.^64−66^ To examine whether Gd-DOTA and Gd-DOTAM-BA are also compatible with DTT, we measured solvent PRE Γ_2_ rates arising from Gd-DOTA or Gd-DOTAM-BA for ubiquitin in a buffer comprised of 20 mM Tris·acetate (pH 7.5), 5% D_2_O, and 5 mM DTT, a concentration of reducing agent that is, in general, higher than that used in most protein NMR experiments. Linear correlation plots of solvent PREs recorded for ^1^H_N_ nuclei of ubiquitin samples with and without 5 mM DTT are illustrated in Figure 7, along with the resultant ϕ_ENS_ potentials (see Figure S8 for the corresponding plots for ^1^H_α_ and methyl ^1^H nuclei). The good agreement between the data obtained with and without DTT indicates, as expected, that the reducing agent has little effect on the fidelity of electrostatic measurements using the positive and negative Gd chelates.

### Application to a Redox-Regulated Molecular Switch

Since reducing agents do not interfere with Gd-based measurements of electrostatic potentials, the range of applications of the NMR technology is greatly increased. In the present study, we illustrate it through an application to electrostatic potentials in the redox-regulated molecular switch within the A-box domain of the HMGB1 protein. HMGB1 functions both in the cell nucleus as well as in the extracellular space.^67^ Extracellular HMGB1 is initially in the reduced state with all cysteine side chains in the thiol form, but the oxidative extracellular environment leads to the formation of a disulfide bond between C23 and C45 of the A-box domain over a period of minutes to hours.^68,69^ The disulfide-bond formation acts as a molecular switch that converts extracellular HMGB1 from a chemoattractant into an inflammatory factor.^67,70^ This switching is associated with an ∼20-fold decrease in affinity for the CXCL12 chemokine and an ∼10-fold increase in affinity for the TLR4·MD-2 receptor complex.^71,72^

**Figure 8:**
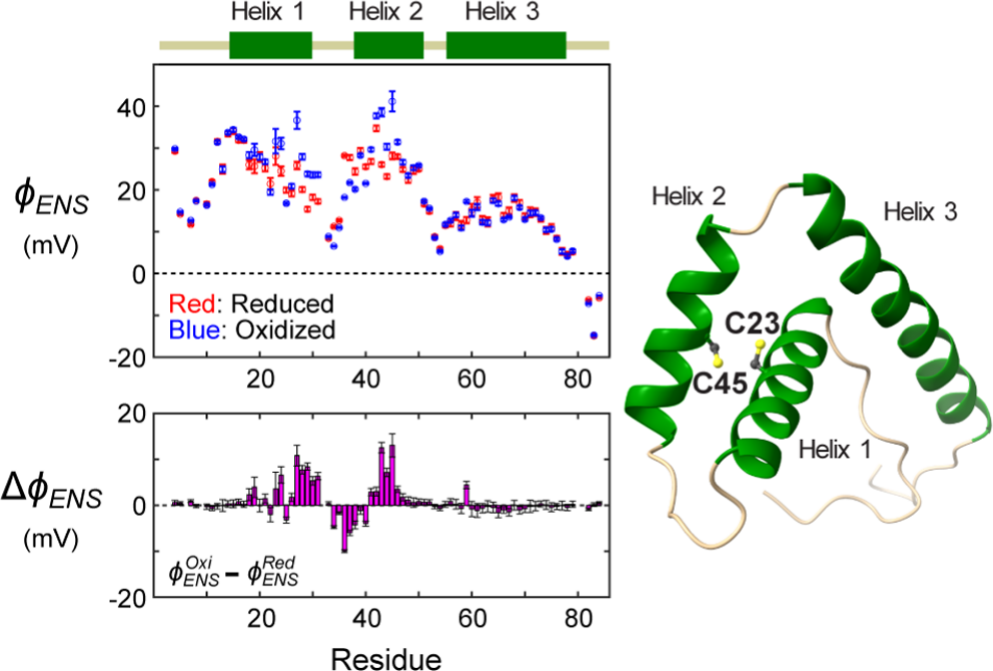
Changes in electrostatics of the redox-regulated molecular switch of the HMGB1 A-box domain as a function of the oxidation status of C23 and C45, revealed through Gd-based measurements of ϕ_ENS_ potentials. The measurement for the reduced state was conducted in the presence of 5 mM DTT to maintain the thiol forms of C23 and C45. In the oxidized state, C23 and C45 form a disulfide bond. The bar graph shows changes in ϕ_ENS_ potentials upon formation of the C23–C45 disulfide bond.

Using the Gd chelates, we measured ϕ_ENS_ values for both the reduced and oxidized states of the HMGB1 A-box domain under identical conditions, with the exception that samples of the reduced protein contained 5 mM DTT. As previously shown, A-box in each redox state exhibits high-quality NMR spectra, which are remarkably different between the two states.^36^Figure 8 compares the ϕ_ENS_ potentials of the reduced and oxidized states. Interestingly, a remarkable change in ϕ_ENS_ upon oxidation was observed for some residues between the oxidation sites of C23 and C45. For example, the ϕ_ENS_ potentials observed for H27 H_N_ changed from 26 to 38 mV, for V36 H_N_ from 28 to 18 mV, for K43 H_N_ from 26 to 40 mV, and for C45 H_N_ from 28 to 43 mV. The changes in the near-surface electrostatic potentials are likely caused by the conformational rearrangement of charged side chains or by shifts in protonation/deprotonation equilibria (e.g., for H27 and H31). The Gd-based experimental data establish that the molecular switching event is associated with significant changes in electrostatics, an insight that is clearly unavailable from the PROXYL-based method because the nitroxide cosolutes cannot be used with reducing agents.

## Discussion

Over the past three decades, a number of Gd chelates have been developed as contrast agents for magnetic resonance imaging because they exhibit large relaxivities for water ^1^H magnetization.^55^ Gd chelates were also used for rapid and quantitative NMR-based metabolomics.^73,74^ For biological macromolecules, Gd chelates have been used to measure solvent PREs for identifying molecular surfaces and interfaces.^40,41^ Since the seminal work of Pintacuda and Otting in the early 2000s demonstrating that Gd-diethylenetriamine pentaacetic acid bismethylamide (Gd-DPTA-BMA) can be used as a probe for identifying protein surfaces, neutral Gd chelates (Gd-DPTMA-BMA,^56^ Gd-HP-DO3A,^65^ Gd-triethylenetetramine hexaacetate trimethylamide [Gd-TTHA-TMA]^75^) have been used in solvent PRE studies, informing on biomolecular structure and dynamics.^41^ The neutral Gd chelates are thought to be less biased in their spatial distribution relative to charged Gd compounds and, thus, able to probe macromolecular surfaces more accurately.^41,56^ By contrast, our current approach takes advantage of the biased spatial distribution of charged Gd-chelate cosolutes to determine effective near-surface electrostatic potentials.

The advantages of the Gd-based approach in comparison to measurements involving nitroxide compounds have been discussed above. From a practical perspective, the preparation of concentrated stock solutions of the Gd cosolutes (>100 mM) is straightforward, as both Gd-DOTA and Gd-DOTAM-BA are highly soluble in H_2_O. In contrast, the commercially available preparation of aminomethyl-PROXYL is a gel-like substance, and its solubility strongly depends on pH so that preparing a stock solution in this case is more demanding.^20^ Once stocks are prepared, the concentrations of cosolutes must be quantified properly to obtain accurate electrostatic potentials. The Evans method is an excellent choice for establishing the concentrations of stock solutions of each Gd-cosolute, Figure 3A, while peak intensities of fully reduced nitroxide-compounds can be used in a straightforward manner for quantifying PROXYL concentrations.^20^

**Figure 9:**
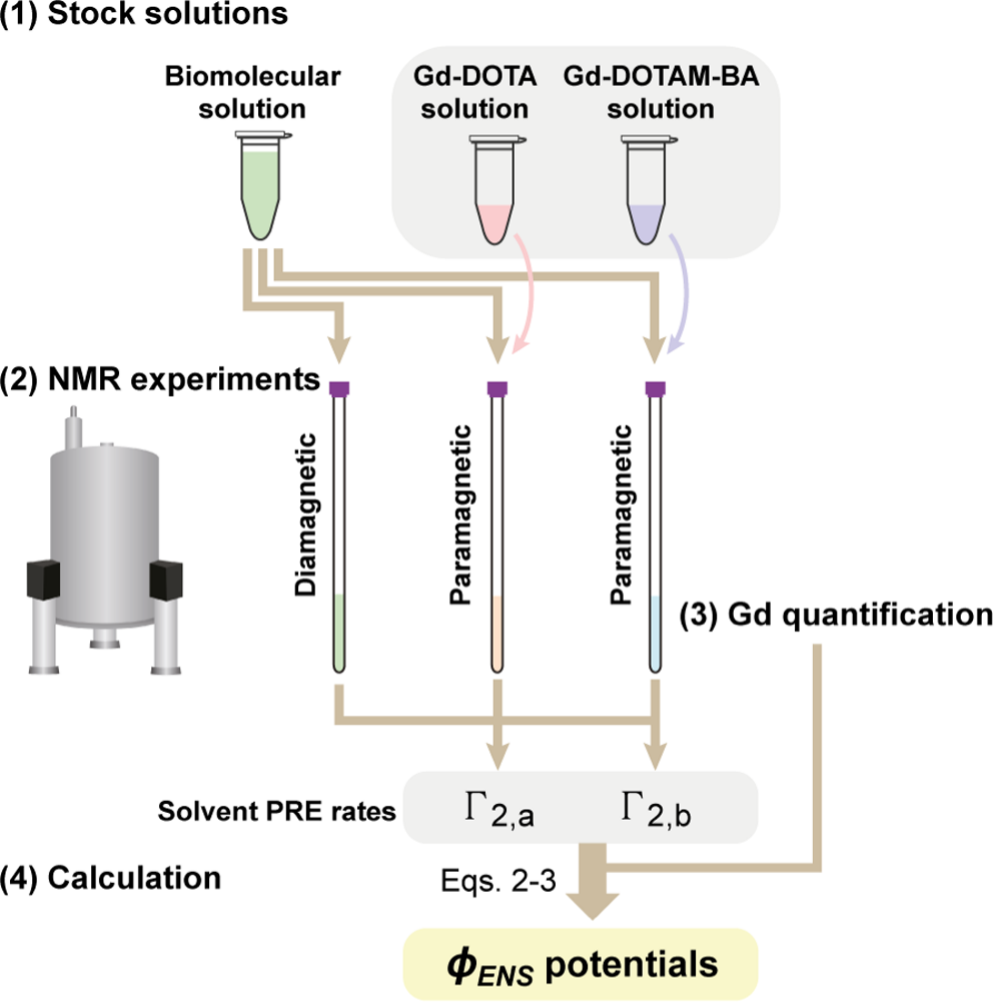
General workflow for the measurement of effective near-surface electrostatic potentials (ϕ_ENS_) of biomolecules using Gd-DOTA (*z*_a_ = −1) and Gd-DOTAM-BA (*z*_b_ = +1).

Figure 9 presents the general workflow for the measurement of ϕ_ENS_ values using Gd-DOTA and Gd-DOTAM-BA, summarized in the following four steps. (1) Preparation of ∼50 to 200 mM stock solutions of cosolutes in water. The Gd concentration in each stock should be measured by the Evans method using solutions diluted (to <10 mM) into a buffer containing molecules that exhibit well-isolated ^1^H signals (e.g., DSS). These stock solutions can be used for many different samples. (2) NMR experiments to measure solvent PRE rates (Γ_2_) for the biomolecule of interest using three samples: a diamagnetic control sample and two paramagnetic samples containing known concentrations of Gd-DOTA or Gd-DOTAM-BA. If the concentrations of the paramagnetic cosolutes are too high, the Γ_2_ decay profiles for observable residues will be of poor quality, while many NMR signals will vanish, and robust measurements of potentials will not be possible. On the other hand, if the concentrations are too low, the PRE rates will be too small to accurately determine ϕ_ENS_ values. A practically useful range of solvent PRE rates Γ_2_ is 3σ < Γ_2_ < 150 s^–1^, where σ is the uncertainty in Γ_2_. Optimal concentrations of cosolutes depend critically on the biomolecule under study; the data shown in Figure 4 can serve as a guide to establish what the appropriate concentrations might be. (3) Establishment of accurate Gd concentrations in biomolecular samples, which is most easily accomplished by measurement of water PREs via ^1^H longitudinal relaxation, in concert with reference profiles of PRE rates vs [paramagnetic cosolute] (as illustrated in Figure 3B). The buffer for the biomolecular sample of interest should also be used to obtain the reference profiles, for which the Gd concentration of each data point is measured by the Evans method. PRE rates measured for the biomolecule of interest are then corrected for concentration differences between cosolutes via eq 3. (4) Calculation of ϕ_ENS_ potentials via eq 2 using the measured Γ_2_ rates, corrected as in (3). The uncertainties in ϕ_ENS_ potentials are estimated from the uncertainties in solvent PRE rates via eq 4. No fitting is involved in the calculation of ϕ_ENS_ potentials from solvent PRE data.

Due to its wide range of applicability and improved performance relative to PROXYL cosolutes, the use of Gd-based methods to measure electrostatic potentials is likely to provide significant insights into biomolecular structure, function, and recognition, in particular for applications that are difficult to investigate using a structure-based computational approach. Examples include electrostatics of single-stranded DNA/RNA, IDR-containing proteins, dynamic multidomain proteins, IDPs, and their complexes with other molecules. For instance, as recently demonstrated,^76^ ϕ_ENS_ potential measurements can reveal how protein domains are influenced by IDRs through electrostatics. The ϕ_ENS_ method is also useful to investigate the potentially critical role of electrostatics in phase separation of IDPs as well.^22,23^ It is likely that measurement of ϕ_ENS_ values can shed light on how post-translational modifications such as phosphorylation and acetylation within IDRs (e.g., histone tails) influence their structural dynamics and hence function. Furthermore, the ϕ_ENS_ data collected for IDPs and IDR-containing proteins may assist the calculation of structural ensembles, in concert with other NMR and small-angle X-ray scattering data.

## Concluding Remarks

We have shown that charged Gd chelates can be used to measure accurate ϕ_ENS_ values for both nucleic acids and proteins, expanding the range of applicability of the NMR approach for studying biomolecular electrostatics. This is particularly the case for proteins that must be maintained in reduced states through the addition of compounds such as DTT or TCEP, which would adversely affect nitroxide radicals but have no effect on their Gd-based counterparts, or for applications where the molecule in question is highly charged so that PREs from one of the PROXYL cosolutes are likely to be small and error prone when single-digit mM concentrations of the PROXYL compounds are used. In contrast, PROXYL derivatives might be preferred in studies of biomolecules that have extraordinarily strong metal binding sites that can capture Gd^3+^ with subpicomolar affinity. It is clear that the availability of Gd-probes represents an important addition to the NMR toolkit for investigating electrostatics with the promise of obtaining invaluable insights into molecular interactions that are critical for biological function.

## Supporting Information Available

Details of structure-based predictions of ϕ_ENS_ potentials; additional experimental data; APBS and DelPhi inputs; the NMR pulse program used to measure water ^1^H *R*_1_ relaxation (Figures S1–S8) (PDF)
