# Supplementary material for: Gadolinium-Based NMR Spin Relaxation Measurements of Near-Surface Electrostatic Potentials of Biomolecules
Source: J Am Chem Soc. 2024 Jul 19;146(30):20788–801. doi: 10.1021/jacs.4c04433 (PMC11295196; doi:10.1021/jacs.4c04433)
Supplement: Supplementary file 1 — ja4c04433_si_001.pdf [file ja4c04433_si_001.pdf]

SUPPORTING INFORMATION

Gadolinium-based NMR spin relaxation measurements of near-surface electrostatic potentials of biomolecules

Binhan Yu, Nicolas Bolik-Coulon, Atul K. Rangadurai, Lewis E. Kay, Junji Iwahara

CONTENTS

Figure S1: Definitions of the parameters involved in Eq. [5]

Figure S2: Optimization of the effective radius r_{pc} of the paramagnetic cosolutes

Figure S3: Examples of spectra recorded for the biomolecules used in the current study

Figure S4: Comparison of ϕ_{ENS} predictions with APBS and DelPhi programs

Figure S5: Explanation for the discrepancy between the experimental and predicted ϕ_{ENS} potentials for ubiquitin L73 H_N

Figure S6: ϕ_{ENS} potentials measured for H_α and methyl ^1H nuclei of ubiquitin using the Gd chelates

Figure S7: ϕ_{ENS} potentials for RtoK CAPRIN1 measured using the positive, neutral and negative Proxyl cosolutes

Figure S8: Impact of 5 mM DTT on solvent PRE rates Γ_2 and ϕ_{ENS} potentials

APBS inputs

DelPhi inputs

NMR pulse program for water ^{1}H R_1 relaxation measurement

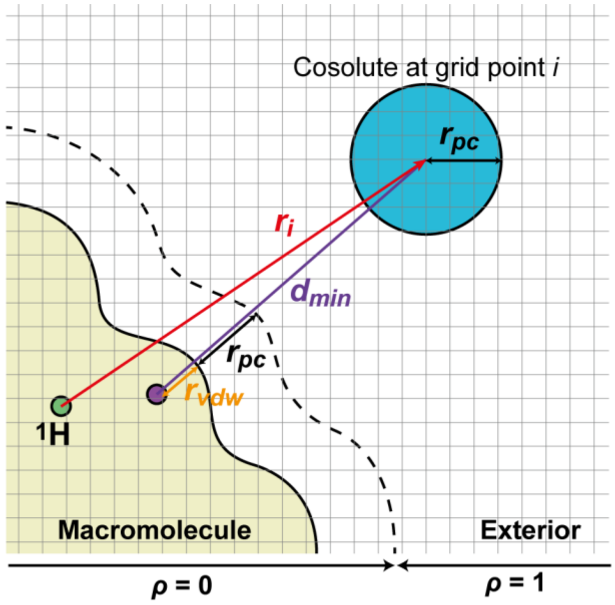

Figure S1. Definitions of the parameters involved in Eq. [5] in the main text. All grid points are used in the evaluation of Eq. [5] where ϕ_i is the electrostatic potential at grid point i (from APBS or DelPhi output); r_i is the distance from the ^{1}H nucleus of interest to a grid point i; and ρ_i is a factor that represents the accessibility of grid point i and is either 1 (accessible) or 0 (inaccessible). A value of 0 was assigned to ρ_i when $d_{min} < r_{vdw} + r_{pc}$, where d_{min} is the distance from grid point i to the closest atom in the macromolecule; r_{vdw} is the van der Waals radius of the closest atom (the value indicated in the PQR-format file); and r_{pc} is the effective radius that defines the accessibility of the paramagnetic center. The interval for the grid space used in calculations was 0.5 Å for each dimension. As shown in Figure S2, r_{pc} = 3.5 Å was obtained for the Gd chelates through empirical optimization. Coincidentally, despite the different chemical structures, r_{pc} = 3.5 Å was also obtained for the PROXYL derivatives in a previous study.[1] It should be noted that r_{pc} is an effective radius and does not represent the molecular radius unless the paramagnetic center is located at the center in a spherical molecule.

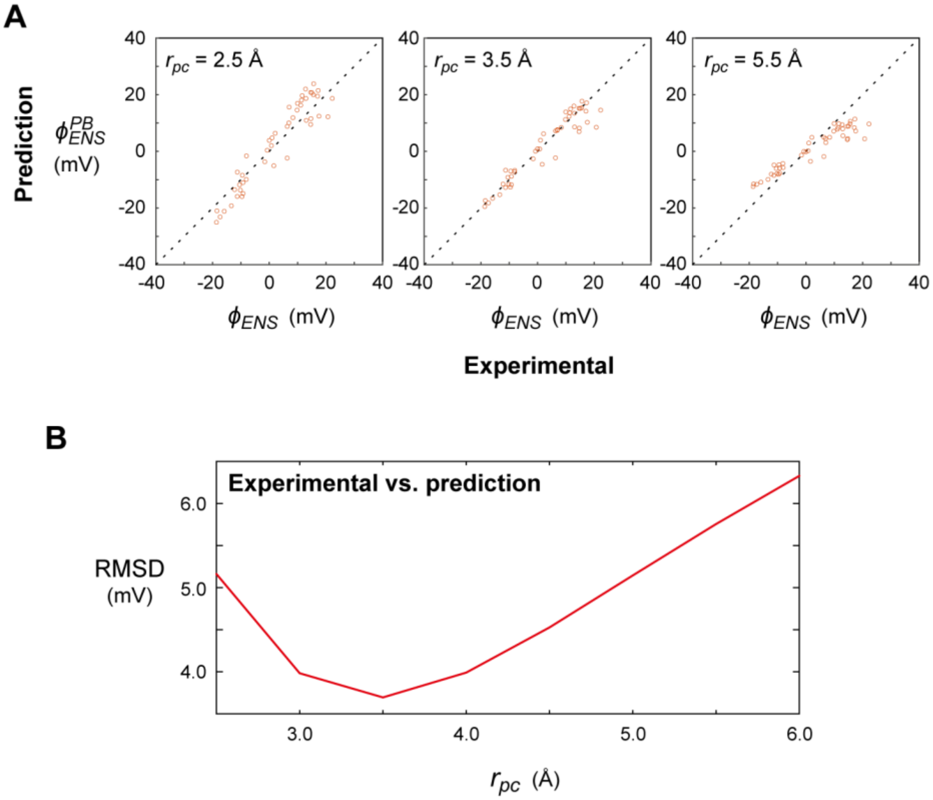

Figure S2. Optimization of the effective radius, r_{pc}, of the Gd-based paramagnetic cosolutes for predicting the effective near-surface electrostatic potential ϕ_{ENS}. (**A**) Correlations between experimental ϕ_{ENS} potentials and Poisson-Boltzmann-based predictions calculated with Eq. [5] for ubiquitin ^1H_N atoms of regions with defined secondary structure. Results with r_{pc} = 2.5, 3.5, and 5.5 Å are shown. (**B**) RMSD between the experimental ϕ_{ENS} potentials and Poisson-Boltzmann-based predictions (Eq. [5]) for ubiquitin ^1H_N atoms in regions of defined secondary structure. An identical value for r_{pc} was assumed for the two analogous compounds Gd-DOTA and Gd-DOTAM-BA. Since the RMSD minimum as a function of r_{pc} is shallow, r_{pc} does not have to be determined more precisely. Based on these results, r_{pc} = 3.5 Å was used for all calculations to predict ϕ_{ENS} potentials in the current study.

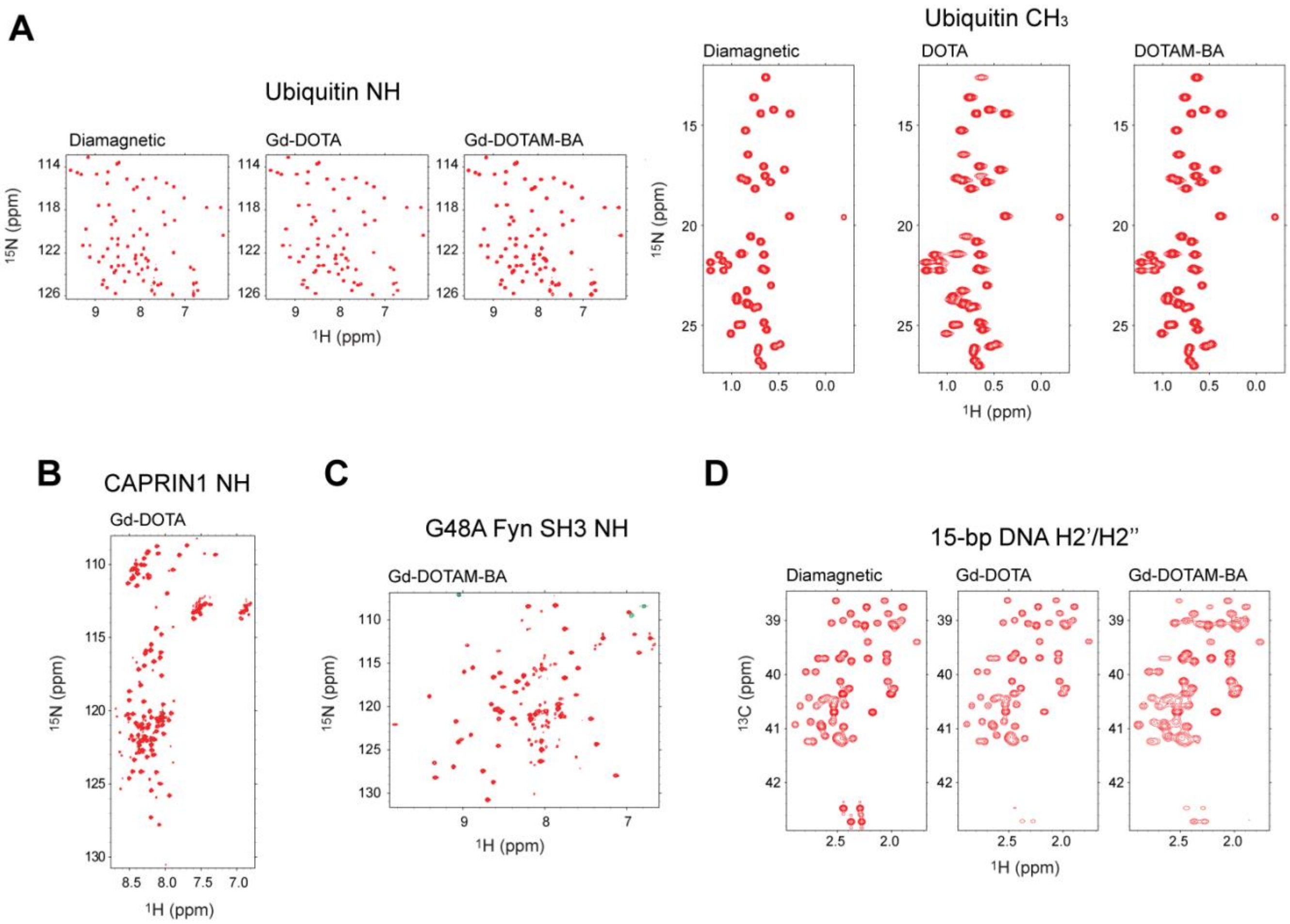

Figure S3. Examples of heteronuclear 2D spectra recorded to measure solvent PRE rates for ubiquitin (**A**), CAPRIN1 (B), G48A Fyn SH3 (C), and 15-bp DNA (D). Some signals are aliased for the ^{15}N and ^{13}C dimensions. The experimental conditions are indicated in the main text.

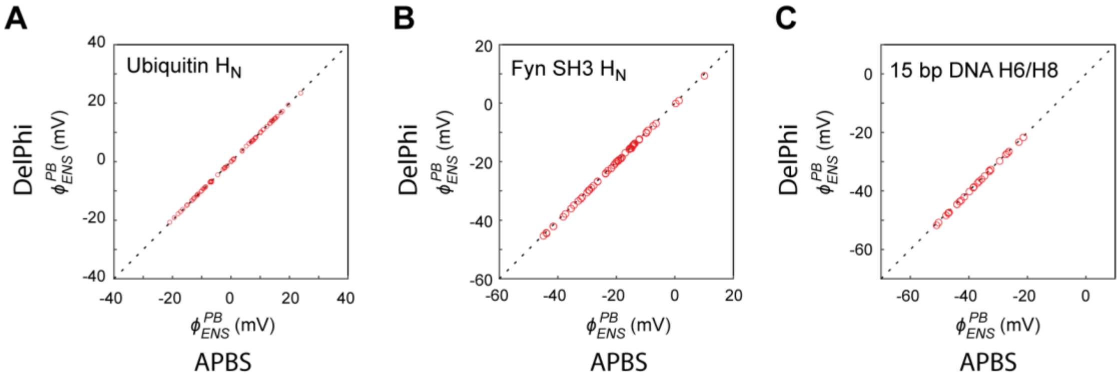

Figure S4. Comparison of structure-based ϕ_{ENS} predictions using electrostatic potentials calculated with APBS[2] and DelPhi[3] programs. (**A**) Ubiquitin at an ionic strength of 24 mM, pH 7.5, and 25˚C. (**B**) G48A Fyn SH3 domain at an ionic strength of 24 mM, pH 6.0, and 10˚C. (**C**) 15-bp DNA at an ionic strength of 123 mM, pH 7.4, and 25˚C. The input parameters for the nonlinear Poisson-Boltzmann based calculations with the APBS and DelPhi programs are given below in the sections "APBS Inputs" and "DelPhi Inputs".

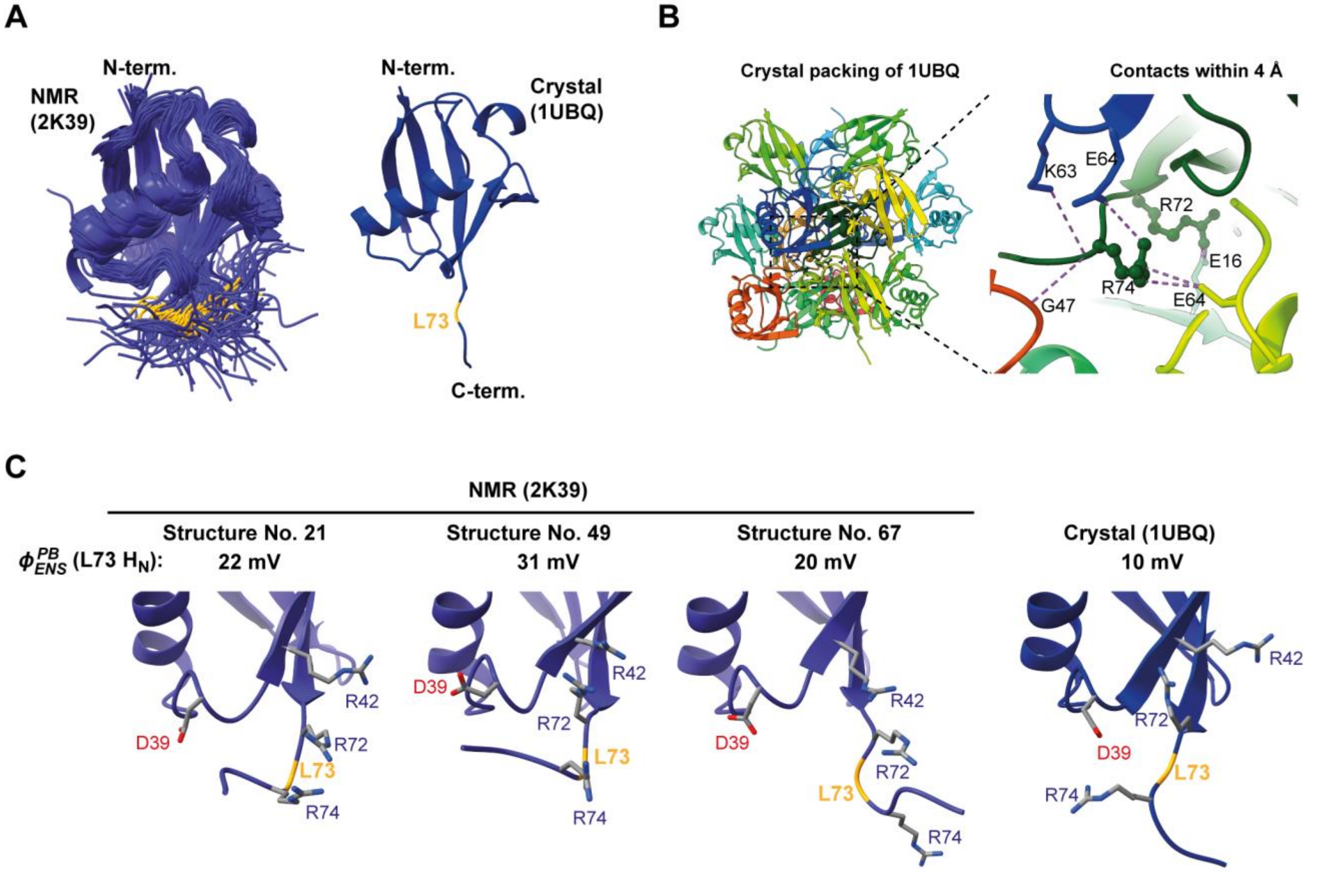

Figure S5 Differences between the flexible C-terminal tail of ubiquitin in solution and the tail immobilized by crystal packing offer an explanation for the discrepancy between the experimental ϕ_{ENS} potential for L73 H_N and that predicted from the crystal structure. (**A**) Ribbon representations of the NMR structures (PDB 2K39)[4] and the crystal structure (PDB 1UBQ)[5]. The structural ensemble of 2K39 includes 116 structures, which are superimposed using the secondary-structure regions. The location of L73 is colored in orange. Note that in solution, the C-terminal tail is disordered, which is also evidenced by small order parameters observed for the NH groups in the C-terminal tail.[4] (**B**) Crystal packing of 1UBQ, showing that the C-terminal tail is fixed by intermolecular contacts. (**C**) Poisson-Boltzmann theory-based prediction of the ϕ_{ENS} potential for L73 H_N for some NMR structures selected from PDB 2K39 and for the crystal structure. The charged side chains whose conformations significantly influence the effective near-surface electrostatic potential (i.e., D39, R42, R72, and R73) are shown. In the crystal structure, for which the predicted ϕ_{ENS} potential of L73 H_N is relatively small, the negatively charged carboxylate group of D39 is pointing toward L73 H_N, whereas the positively charged guanidinium groups of R42, R72, and R74 are pointing away from L73 H_N.

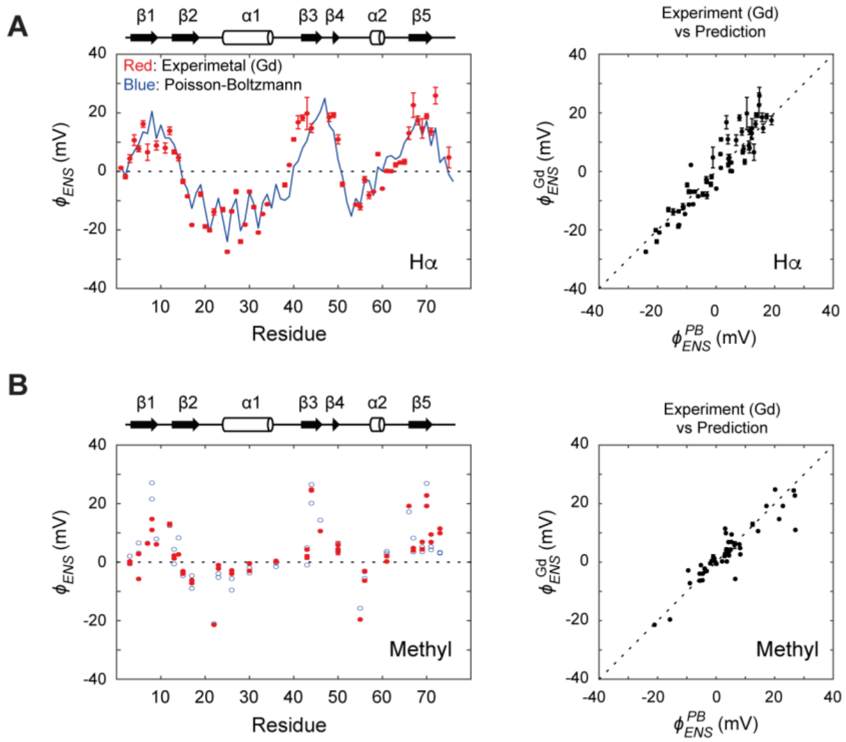

Figure S6. Effective near-surface electrostatic potentials ϕ_{ENS} measured for H$_\alpha$ and methyl ^{1}H nuclei of ubiquitin using Gd-DOTA and Gd-DOTAM-BA cosolutes. The experimental data were compared with the predictions from the Poisson-Boltzmann electrostatic potentials. The RMSDs between the experimental values and the predictions were 4.8 mV for H$_\alpha$ atoms and 3.2 mV for methyl groups in the regions of defined secondary structure.

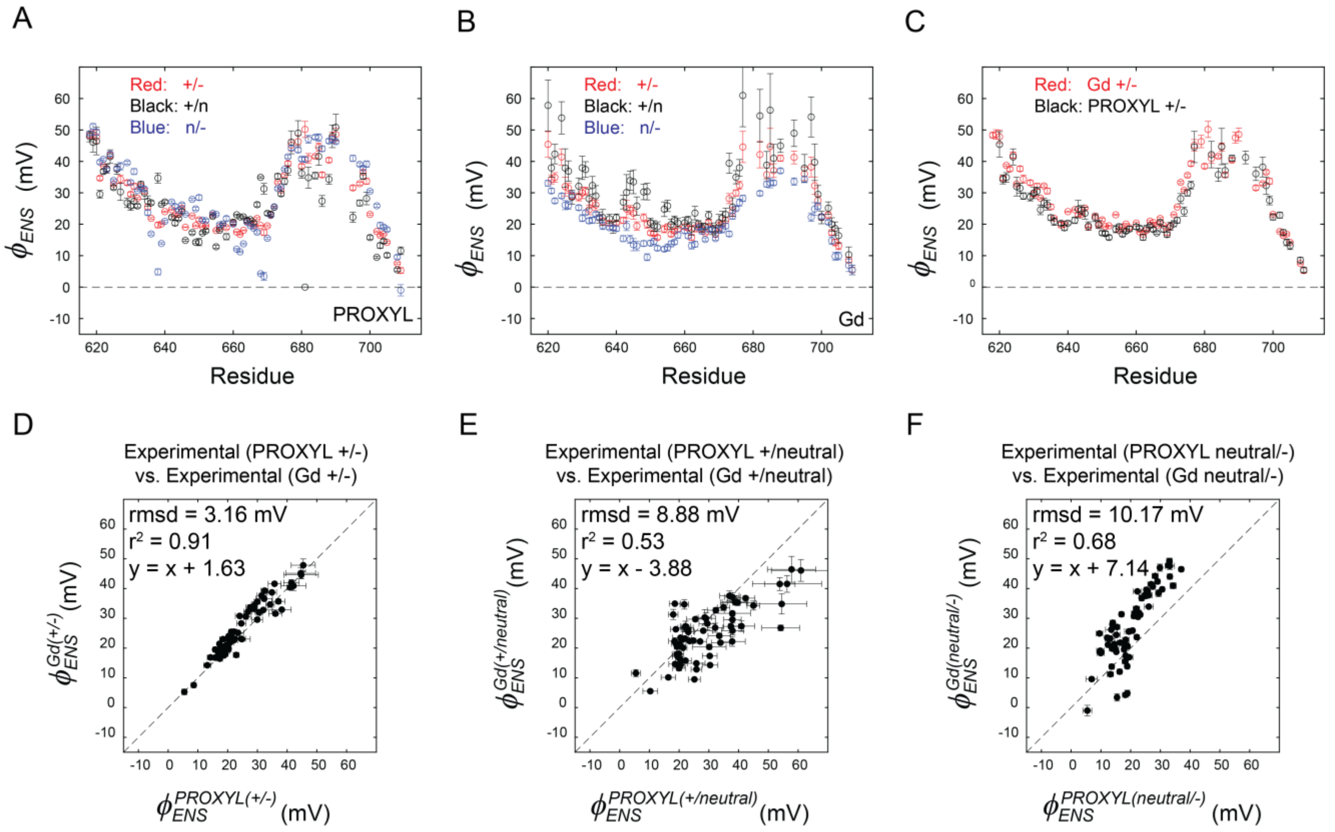

Figure S7. ϕ_{ENS} potentials measured for ^1H_N nuclei of RtoK CAPRIN1 using neutral paramagnetic cosolutes. (A) Overlaid ϕ_{ENS} potentials measured using +/-, +/neutral and neutral/- pairs of PROXYL compounds. (B) Overlaid ϕ_{ENS} potentials measured using +/-, +/neutral and neutral/- pairs of Gd-chelates. (C) Comparison of ϕ_{ENS} potentials measured from +/- pairs of PROXYL and Gd cosolutes. (D) Correlation of ϕ_{ENS} potentials measured using +/- pairs of PROXYL and Gd cosolutes. (E) Correlation of ϕ_{ENS} potentials measured using the +/neutral pairs of PROXYL- and Gd-based compounds. (F) As in D and E but using the neutral/- pairs of cosolutes.

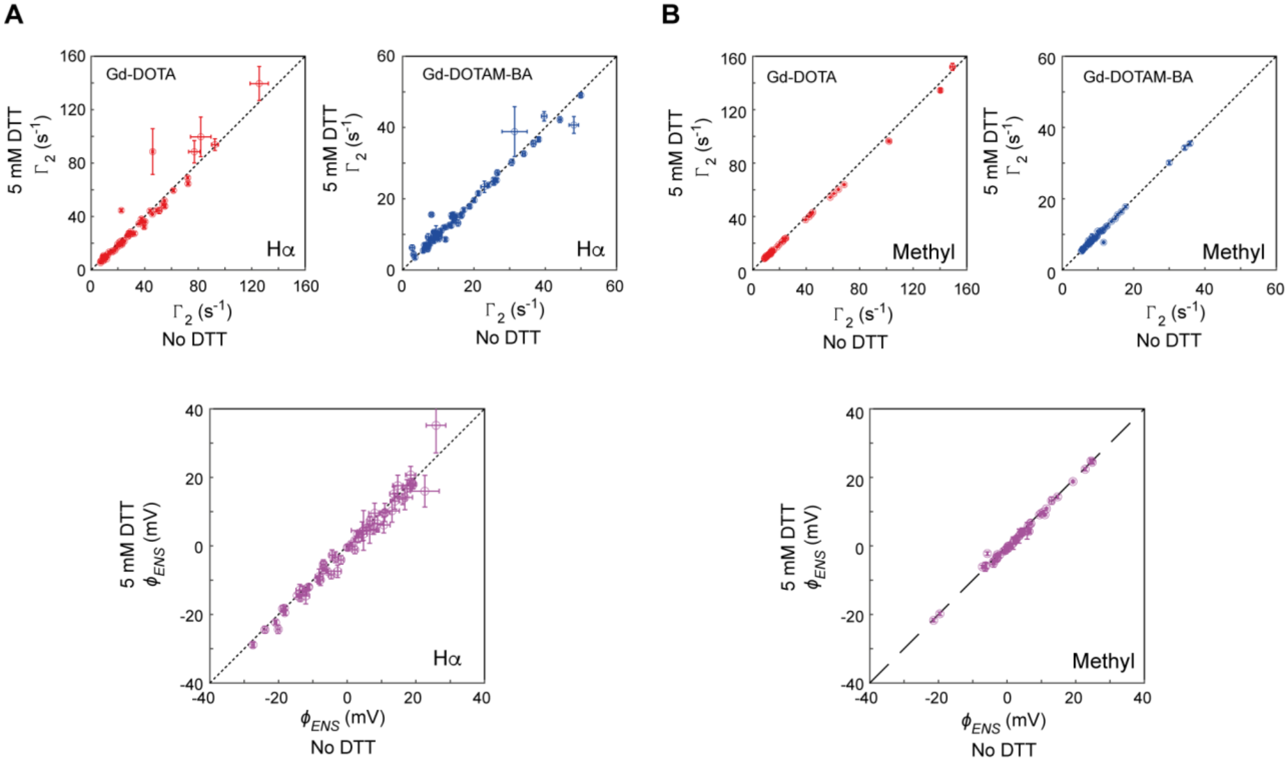

Figure S8. Impact of 5 mM DTT on solvent Γ_2 PRE rates (top) and on the ϕ_{ENS} potentials (bottom) measured for H_α (panel **A**) and methyl ^{1}H nuclei (panel **B**) of ubiquitin using Gd-DOTA and Gd-DOTAM-BA as paramagnetic cosolutes. The solution conditions were the same as those for Figure 6.

1. APBS inputs

The following inputs were used for nonlinear Poisson-Boltzmann equation-based calculations with APBS[2]. The output files from APBS in the “dx” format were used to predict the effective near-surface potentials ϕ_{ENS} using Eq. [5]. The MATLAB script package ‘PBENS’, which is available at a GitHub webpage (https://github.com/IwaharaLab/PBENS), was used for the calculations.

1a. APBS input to calculate electrostatic potentials of ubiquitin

```
read
    mol pqr 1ubq.pqr
end
elec
    mg-auto
    dime 257 257 257
    cglen 138 138 138
    fglen 128 128 128
    cgcent mol 1
    fgcent mol 1
    mol 1
    npbe
    bcfl sdh
    pdie 2.0000
    sdie 78.5400
    ion charge  1.000 conc 0.024 radius 2.000
    ion charge -1.000 conc 0.024 radius 2.000
    srfm smol
    chgm spl2
    sdens 10.00
    srad 1.40
    swin 0.30
    temp 298.15
    calcenergy total
    calcforce no
    write pot dx  1ubq_ic24mM_pot2.0
end
print elecEnergy 1 end
quit
```

The PQR file, 1ubq.pqr, was generated from the PDB file1ubq using the PDB2PQR program[6] along with PROPKA[7]-based selection of titration states at pH 7.5 and the AMBER force field parameters. The ion concentrations (0.024 M) and the temperature (298.15 K) were set based on the experimental conditions. The 3D space is 128 Å × 128 Å × 128 Å with an interval of 0.5 Å (257 points along each dimension). The dielectric constants were set to 2.0 for the interior of the protein and to 78.54 for the solvent.

1b. APBS input to calculate electrostatic potentials of G48A Fyn SH3

```
read
    mol pqr 3cqt_mutate2.pqr
end
elec
    mg-auto
    dime 257 257 257
    cglen 138 138 138
    fglen 128 128 128
    cgcent mol 1
    fgcent mol 1
    mol 1
    npbe
    bcfl sdh
    pdie 2.0000
    sdie 78.5400
    ion charge  1.000 conc 0.024 radius 2.000
    ion charge -1.000 conc 0.024 radius 2.000
    srfm smol
    chgm spl2
    sdens 10.00
    srad 1.40
    swin 0.30
    temp 283.15
    calcenergy total
    calcforce no
    write pot dx  3cqt_mutate2_ic24mM_pot2.0
end
print elecEnergy 1 end
quit
```

The PQR file, 3cqrt_mutate2.pqr, was generated from a PDB-format file using the PDB2PQR program[6] along with PROPKA[7]-based selection of titration states at pH 6.0 and the AMBER force field parameters. The ion concentrations (0.024 M) and the temperature (283.15 K) were set based on the experimental conditions. The 3D space is 128 Å × 128 Å × 128 Å with an interval of 0.5 Å (257 points along each dimension). The dielectric constants were set to 2.0 for the interior of the protein and to 78.54 for the solvent.

1c. APBS input to calculate electrostatic potentials of the 15-bp DNA

```
read
    mol pqr eg15.pqr
end
elec
    mg-auto
    dime 321 321 321
    cglen 180 180 180
    fglen 160 160 160
    cgcent mol 1
    fgcent mol 1
    mol 1
    npbe
    bcfl sdh
    pdie 2.0000
    sdie 78.5400
    ion charge 1.000 conc 0.123 radius 2.000
    ion charge -1.000 conc 0.123 radius 2.000
    srfm smol
    chgm spl2
    sdens 10.00
    srad 1.40
    swin 0.30
    temp 298.15
    calcenergy total
    calcforce no
    write pot dx  eg15_pot2.0
end
print elecEnergy 1 end
quit
```

The PQR file, eg15.pqr, was generated from a PDB-format file using the PDB2PQR program[6] along with PROPKA[7]-based selection of titration states at pH 7.4 and the AMBER force field parameters. The ion concentrations (0.123 M) and the temperature (298.15 K) were set based on the experimental conditions. The 3D space is 160 Å × 160 Å × 160 Å with an interval of 0.5 Å (321 points along each dimension). The dielectric constants were set to 2.0 for the interior of DNA and to 78.54 for the solvent.

2. Delphi inputs

The following inputs were used for nonlinear Poisson-Boltzmann equation-based calculations with DelPhi[3]. The output files from DelPhi in the "cube" format were used to predict the effective near-surface potentials ϕ_{ENS} using Eq. [5]. The MATLAB script package 'PBENS' was used for the calculations.

2a. DelPhi input to calculate electrostatic potentials of ubiquitin

```
gsize=257
scale=2.0
temperature=25
out(phi,file=1ubq_ic24mM_pot_2.0.cube,format=cube)
in(modpdb4,file="1ubq_forDelphi.pqr",format=pqr)
Center(unit=15)
indi=2.0
exdi=78.54
prbrad=1.4
salt=0.024
ionrad=2.0
bndcon=2
maxc=0.0001
nonit=100
energy(s,c,g)
```

The ion concentrations (0.024 M) and the temperature (25˚C) were set based on the experimental conditions. The 3D space is 128 Å × 128 Å × 128 Å with an interval of 0.5 Å (257 points along each dimension). The dielectric constants were set to 2.0 for the interior of the protein and to 78.54 for the solvent.

2b. DelPhi input to calculate electrostatic potentials of G48A Fyn SH3

```
gsize=257
scale=2.0
temperature=10
out(phi,file=3cqt_mutate2_ic24mM_pot_2.0.cube,format=cube)
in(modpdb4,file="3cqt_mutate2_forDelphi.pqr",format=pqr)
Center(unit=15)
indi=2.0
exdi=78.54
prbrad=1.4
salt=0.024
ionrad=2.0
bndcon=2
maxc=0.0001
nonit=100
energy(s,c,g)
```

The ion concentrations (0.024 M) and the temperature (10˚C) were set based on the experimental conditions. The 3D space is 128 Å × 128 Å × 128 Å with an interval of 0.5 Å (257 points along each dimension). The dielectric constants were set to 2.0 for the interior of the protein and to 78.54 for the solvent.

2c. DelPhi input to calculate electrostatic potentials of the 15-bp DNA

```
gsize=321
scale=2.0
temperature=25
out(phi,file=eg15_pot_2.0.cube,format=cube)
in(modpdb4,file="eg15_forDelphi.pqr",format=pqr)
Center(unit=15)
indi=2.0
exdi=78.54
prbrad=1.4
salt=0.123
ionrad=2.0
bndcon=2
maxc=0.0001
nonit=100
energy(s,c,g)
```

The ion concentrations (0.123 M) and the temperature (25˚C) were set based on the experimental conditions. The 3D space is 160 Å × 160 Å × 160 Å with an interval of 0.5 Å (321 points along each dimension). The dielectric constants were set to 2.0 for the interior of DNA and to 78.54 for the solvent.

3. NMR pulse program for water ^{1}H R_1 measurement

Measurement of water ^{1}H R_1 relaxation with a high-field NMR instrument is generally nontrivial due to radiation damping. Although probe detuning has been used to suppress radiation damping in a trade-off against signal-to-noise,[8] the extent of detuning required for accurate measurement may not be obvious to researchers. The following pulse program for Bruker NMR spectrometers was designed to measure water ^{1}H R_1 relaxation rates without the requirement of detuning. This pulse program was used to obtain the water relaxivity data shown in Figure 3B. Note that the net longitudinal relaxation time is time_relax + 2u + p55 + d16 + 4u.

```
/* 1HT1_water_lek_500_cp

  Used to record 1H T1 of water as a series of 1Ds

  Written by LEK May 22, 2016

  Magnetization originates as zero and measure build up (1- exp(-T/T1) profile

  Modified for cryo probe

*/

#include <Avance.incl>
#include <Grad.incl>
#include <Delay.incl>

;Define phases
#define zero ph=0.0
#define one ph=90.0
#define two ph=180.0
#define three ph=270.0

;Define Pulses
define pulse pwh
       "pwh=p1"             ; 1H hard pulse at power pl1

define pulse pwh_theta
       "pwh_theta=p12"             ; small tip angle pulse

;Define delays

"in0=inf1/2"
"d11=30m"

define delay dly_lk
  "dly_lk = d17"

define list<delay> time_relax = <$VDLIST>

 "cnst12 = plw12"  ; power level for 1H scrambling pulse

"l2 = 0"  ; pointer to vd list for magnetization T1 recovery

1 ze
```

```
; check validity of parameters

  if "cnst12 > 4.0"
  {
   2u
   print "error: power level for 1H locking is too large < 4W"
   goto HaltAcqu
  }

 if "dly_lk > 41m" {
   2u
   print "error: ly_lk too lone < 40 ms"
   goto HaltAcqu
  }

2 d11 do:f2

; continue to check run time variables

"DELTA = time_relax[l2]"

if "DELTA > 20s" {
  2u
  print "error: time_relax is too long < 20s"
  goto  HaltAcqu
}

 2u pl1:f1                        ; power(tpwr)
 d1                               ; delay(d1)

 20u UNBLKGRAD             ; dly 20u, unblank gradients and lock hold

; dephase initial proton magnetization

 2u pl12:f1
 (2u cw zero):f1
 dly_lk                  ; turn on cw decoupling for a delay of dly_lk
 2u do:f1

 2u pl12:f1
 (2u cw one):f1
 "DELTA = dly_lk/2.0"
 DELTA                  ; turn on cw decoupling for a delay of dly_lk
 2u do:f1

  2u
  p55:gp5*0.5       ; gradient 5 * 0.5
  d16

 2u pl1:f1
 (pwh zero):f1

  2u
  p55:gp5       ; gradient 5
  d16

 (pwh one):f1
```

```
  2u
  p55:gp5*0.3       ; gradient 5
  d16

  4u BLKGRAD

 "DELTA = time_relax[l2]"
   DELTA

 (pwh_theta ph1):f1

  go=2 ph31         ; acquire fid
  d11  mc #0 to 2 F0(zd)   ; write FID to disk
  F1QF(calclc(l2,1))

HaltAcqu, 1m
exit

ph1=0 1 2 3
ph31=0 1 2 3
ph26=0
ph27=1
ph28=2
ph29=3

;d1 : repetition delay
;d11 : delay for disk i/o, 30ms
;d16 : gradient recovery delay, 200us
;d17 : delay for 1H scrambling - set to 40 ms
;pl1 : tpwr - power level for pwh
;pl12 : power level for 1H scrambling , typically about 23dB less power than high power
;cnst12 : power in
;p1 : pwh
;p12 : pwh_theta , small tip angle pulse
;zgoptns :
```

References for Supporting Information

(1) Yu, B.; Pletka, C. C.; Pettitt, B. M.; Iwahara, J. De novo determination of near-surface electrostatic potentials by NMR. *Proc Natl Acad Sci U S A* **2021**, *118*, e2104020118.
(2) Jurrus, E.; Engel, D.; Star, K.; Monson, K.; Brandi, J.; Felberg, L. E.; Brookes, D. H.; Wilson, L.; Chen, J.; Liles, K. *et al.* Improvements to the APBS biomolecular solvation software suite. *Protein Sci* **2018**, *27*, 112.
(3) Li, C.; Jia, Z.; Chakravorty, A.; Pahari, S.; Peng, Y.; Basu, S.; Koirala, M.; Panday, S. K.; Petukh, M.; Li, L. *et al.* DelPhi Suite: New Developments and Review of Functionalities. *J Comput Chem* **2019**, *40*, 2502.
(4) Lange, O. F.; Lakomek, N.-A.; Farès, C.; Schröder, G. F.; Walter, K. F. A.; Becker, S.; Meiler, J.; Grubmüller, H.; Griesinger, C.; de Groot, B. L. Recognition Dynamics Up to Microseconds Revealed from an RDC-Derived Ubiquitin Ensemble in Solution. *Science* **2008**, *320*, 1471.
(5) Vijay-Kumar, S.; Bugg, C. E.; Cook, W. J. Structure of ubiquitin refined at 1.8 A resolution. *J Mol Biol* **1987**, *194*, 531.
(6) Dolinsky, T. J.; Czodrowski, P.; Li, H.; Nielsen, J. E.; Jensen, J. H.; Klebe, G.; Baker, N. A. PDB2PQR: expanding and upgrading automated preparation of biomolecular structures for molecular simulations. *Nucleic Acids Res* **2007**, *35*, W522.
(7) Olsson, M. H.; Sondergaard, C. R.; Rostkowski, M.; Jensen, J. H. PROPKA3: consistent treatment of internal and surface residues in empirical pK_a predictions. *J Chem Theory Comput* **2011**, *7*, 525.
(8) Krishnan, V. V.; Murali, N. Radiation damping in modern NMR experiments: Progress and challenges. *Prog Nucl Magn Reson Spectrosc* **2013**, *68*, 41.
